# Local Environments Created by the Ligand Coating of Nanoparticles and Their Implications for Sensing and Surface Reactions

**DOI:** 10.1021/acs.accounts.3c00139

**Published:** 2023-08-22

**Authors:** Florian Schulz, Jonas Hühn, Marco Werner, Dominik Hühn, Julia Kvelstad, Ulrich Koert, Nicole Wutke, Markus Klapper, Michael Fröba, Vladimir Baulin, Wolfgang J. Parak

**Affiliations:** 1 Fachbereich Physik, Universität Hamburg, 22607 Hamburg, Germany; 2 Fachbereich Physik, Philipps Universität Marburg, 35037 Marburg, Germany; 3 Leibniz-Institut fur Polymerforschung Dresden e.V., 01069 Dresden, Germany; 4 Fachbereich Chemie, Philipps Universität Marburg, 35043 Marburg, Germany; 5 Max Planck Institute für Polymerforschung, 55128 Mainz, Germany; 6 Fachbereich Chemie, Universität Hamburg, 20146 Hamburg, Germany; 7 Departament Quimica Fisica i Inorganica, Universitat Rovira i Virgili, 43007 Tarragona, Spain

## Abstract

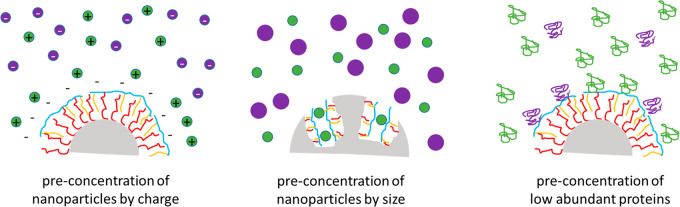

The ligand shells of colloidal nanoparticles (NPs) can serve different purposes. In general, they provide colloidal stability by introducing steric repulsion between NPs. In the context of biological applications, the ligand shell plays a critical role in targeting, enabling NPs to achieve specific biodistributions. However, there is also another important feature of the ligand shell of NPs, namely, the creation of a local environment differing from the bulk of the solvent in which the NPs are dispersed. It is known that charged ligand shells can attract or repel ions and change the effective charge of a NP through Debye–Hückel screening. Positively charged ions, such as H^+^ (or H_3_O^+^) are attracted to negatively charged surfaces, whereas negatively charged ions, such as Cl^–^ are repelled. The distribution of the ions around charged NP surfaces is a radial function of distance from the center of the NP, which is governed by a balance of electrostatic forces and entropy of ions and ligands. As a result, the ion concentration at the NP surface is different from its bulk equilibrium concentration, i.e., the charged ligand shell around the NPs has formed a distinct local environment. This not only applies to charged ligand shells but also follows a more general principle of induced condensation and depletion. Polar/apolar ligand shells, for example, result in a locally increased concentration of polar/apolar molecules. Similar effects can be seen for biocatalysts like enzymes immobilized in nanoporous host structures, which provide a special environment due to their surface chemistry and geometrical nanoconfinement. The formation of a local environment close to the ligand shell of NPs has profound implications for NP sensing applications. As a result, analyte concentrations close to the ligand shell, which are the ones that are measured, may be very different from the analyte concentrations in bulk. Based on previous work describing this effect, it will be discussed herein how such local environments, created by the choice of used ligands, may allow for tailoring the NPs’ sensing properties. In general, the ligand shell around NPs can be attractive/repulsive for molecules with distinct properties and thus forms an environment that can modulate the specific response. Such local environments can also be optimized to modulate chemical reactions close to the NP surface (for example, by size filtering within pores) or to attract specific low abundance proteins. The importance hereby is that this is based on interaction with low selectivity between the ligands and the target molecules.

## Key References

- ZhangF.; AliZ.; AminF.; FeltzA.; OheimM.; ParakW. J.Ion and pH sensing with colloidal nanoparticles: influence of surface charge on sensing and colloidal properties. ChemPhysChem2010, 11, 730–7352013566810.1002/cphc.200900849.^1^*This article shows how Debye–Hückel screening increases the H^+^ concentration close to negatively charged surfaces, i.e., decreases the local pH, and how the distance dependence of this process can be experimentally investigated*.
- RiedingerA.; ZhangF.; DommershausenF.; RöckerC.; BrandholtS.; NienhausG. U.; KoertU.; ParakW. J.Ratiometric Optical Sensing of Chloride Ions with Organic Fluorophore - Gold Nanoparticle Hybrids: A Systematic Study of Distance Dependency and the Influence of Surface Charge. Small2010, 6, 2590–25972095776010.1002/smll.201000868.^2^*Here the opposite direction is shown: how the local Cl^–^ concentration close to a negatively charged surface is decreased by Debye–Hückel screening*.
- FriedD. I.; TroppK.; FröbaM.On the Way to Cofactor Regeneration in Nanopores: Tailoring Porous Materials for Glucose-6-phosphate Dehydrogenase Immobilization. ChemCatChem.2013, 5, 931–938.^3^*In this article, consequences for reactions in confined spaces are described*.

## Introduction

Colloidal nanoparticles (NPs) can react with their environment. For this, accumulation of the reaction partner on the NP surface is needed, which is controlled by the NP ligand shell. In general, the reaction between the NP and the targeted reaction partner should be highly specific, and for this, the binding of the targeted reaction partner to the NP surface should be highly selective. To give an example, in the case that a NP is used as a label for a target protein on the surface of cells, the NP can be modified with ligands, such as antibodies, which should bind as selectively as possible to this target protein, i.e., they should bind as little as possible to other proteins on the cell surface. Thus, generally, when certain molecules from the NP’s environment should bind to the NP surface, the NP surface will be modified with ligands that are highly selective for these molecules. In this Account, we want to make the argument that such a design strategy aiming for ligands with high selectivity can be supplemented (or even replaced) by ligands with low selectivity, as will be discussed for three examples. The first example deals with ion-responsive fluorophores immobilized at the NP surface. Such ion-responsive fluorophores should bind as selectively to their target ions as possible. However, the attraction between the ion-responsive fluorophores and their target ions in general has a very short-range. By adding ligands to the NP surface which attract the target ions over a longer range (i.e., by electrostatic interaction), which however will be possible only with lower selectivity, the target ions would be accumulated close to the NP surface, where they then could bind to the ion-sensitive fluorophores with high selectivity. The involvement of ligands with low selectivity but longer working range thus would help to preconcentrate the target ions (and also similar ions), and the final selective binding of the target ions then would be accomplished on short-range with the ion-responsive fluorophores. The second example deals with modulation of the reaction space. Let us image a specific reaction on the surface of a NP, for example, by enzymes linked to the surface of NPs that selectively process substrate molecules from the surrounding solution. Hereby the enzyme acts with high selectivity on its respective target substrates. Also such a reaction can be tuned by introducing ligands with low selectivity. If the NPs are porous and the enzymes would be linked in the pores, ligands of different length could tune the size of the pores and thus regulate entry of substrate molecules based on their size. Such “preselection” of access based on size would have only low selectivity, but it would be an additional “filter” as gatekeeper to the place where the actual specific reaction (in our example the enzyme substrate reaction) takes place. Ligands can also change the reaction conditions, for example, the local pH, which can also influence, for example, enzymatic reactions. Again, this is not very selective regarding the particular enzyme substrate reaction but helps to drive the reaction. As a third example, the adsorption of proteins to the surface of NPs will be discussed, which is largely controlled by the ligands present on the NP surface. If one analyzes the composition of proteins adsorbed to a NP surface, for example, by mass spectrometry, it will be different than the protein composition in the surrounding medium. NPs can be thus used to “fish” for low abundance proteins. In the case that only one type of protein should be extracted, a stealth NP surface that suppresses nonspecific adsorption would be modified with an antibody as a ligand against the target protein, in order to achieve the best selectivity in the binding of this particular target protein. However, this NP would be very limited; it could extract only one type of protein. We will discuss how instead, by using ligands with low selectivity, NPs can be used to enrich different libraries of proteins on their surface. These three examples demonstrate what benefits a ligand shell that attracts molecules from the environment with low selectivity can have.

## Tuning the Working Point of Ion-Responsive Fluorophores by Different Ligand Coatings of NPs

**Figure 1:**
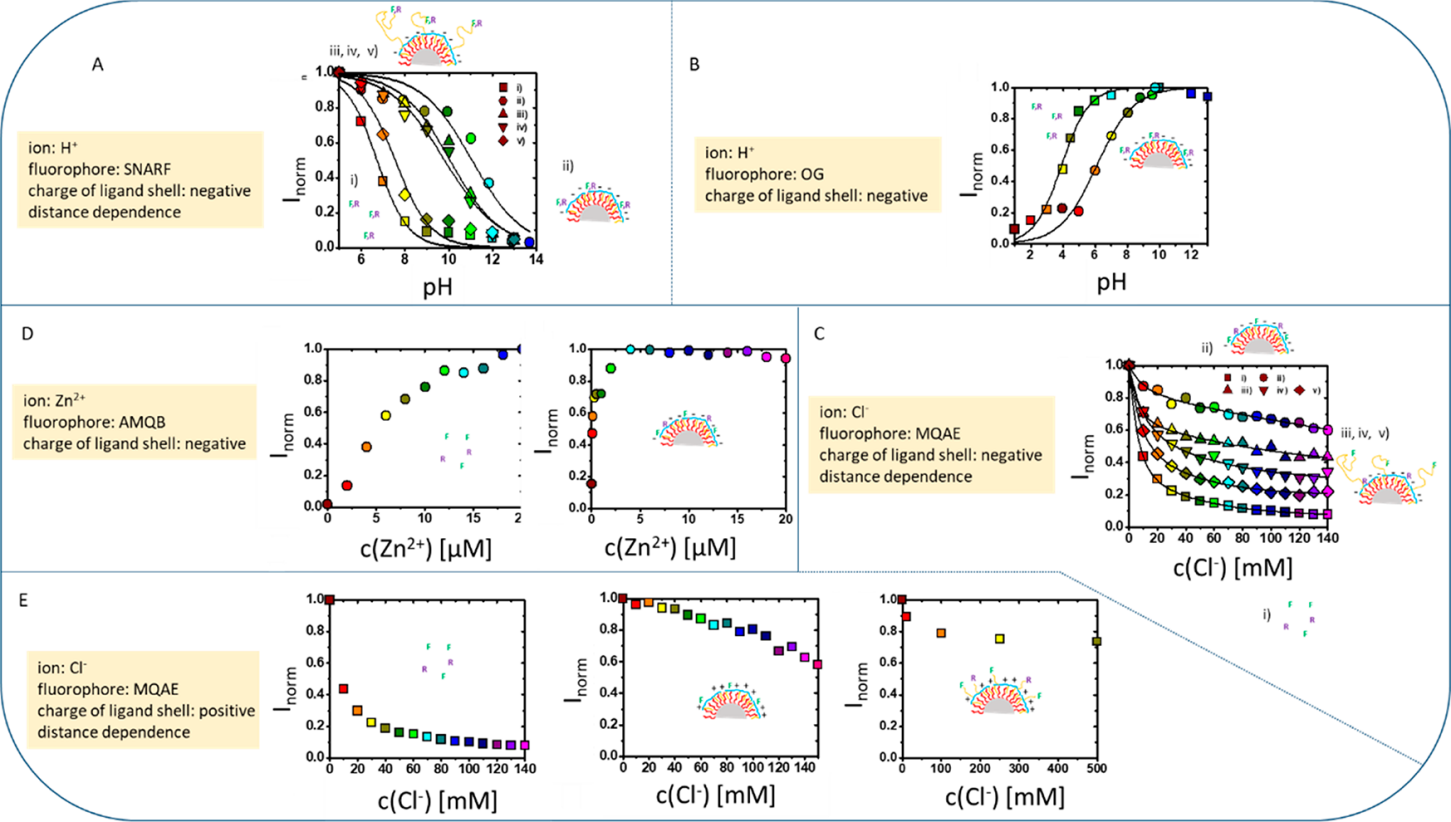
Tuning of the fluorescence response of ion responsive organic fluorophores “F” (drawn in green), which are immobilized at the surface of charged NPs. In order to allow for ratiometric measurements at the same time also nonresponsive reference fluorophores “R” (drawn in violet) are attached to the NP surface. The negative “–” or positive “+” charge of the NP surface is provided by an amphiphilic polymer coating (drawn in red and blue) around the NP surface (NP core drawn in gray; organic ligand capping drawn in yellow). The distance between the fluorophores and the NP surface is optionally varied by the introduction of molecular spacers (shown in yellow). The graphs show the normalized ratiometric fluorescence read out *I*_norm_ versus the ion concentration *c*. (A) Seminaphtharhodafluor (SNARF)^14^ was used as a pH-responsive fluorophore F. The distance between SNARF and the negatively charged polymer surface was varied with PEG molecules of different molecular weight. This graph is adopted with permission from Zhang et al.^1^ (see the SI). Copyright 2010 John Wiley and Sons. (B) Oregon green (OG)^15^ was used as pH-responsive fluorophore F attached to a negatively charged polymer surface. This graph is adopted with permission from Zhang et al.^11^ (see the SI). Copyright 2011 John Wiley and Sons. (C) 2-[2-(6-methoxyquinolinium chloride)ethoxy]-ethanamine hydrochloride (MQAE)^16^ was used as Cl^–^-responsive fluorophore F. The distance between MQAE and the negatively charged polymer surface was varied with PEG spacers of different molecular weight. This graph is adopted with permission from Riedinger et al.^2^ (see the SI). Copyright 2010 John Wiley and Sons. (D) 4-Aminomethyl-*N*-(6-methoxy-quinolin-8-yl)-benzenesulfonamide (AMQB)^17^ was used as Zn^2+^-responsive fluorophore F, attached to a negatively charged polymer surface. For the experimental details of these (so far unpublished) data, see the SI. (E) MQAE was attached to the surface of a positively charged polymer, and the distance was optionally varied with PEG. For the experimental details of these (so far unpublished) data, see the SI.

Fluorescent NPs are frequently used for the purpose of ion-sensing.^4−6^ There are numerous reports on how the presence of certain ions may change the fluorescence properties of NPs. This can be manifested in intrinsically fluorescent NPs, such as quantum dots, whereby the presence of ions can quench or enhance fluorescence,^7,8^ either by direct interaction with the NP surface^9^ or by more elaborated mechanisms involving a combination of NPs and other NPs or molecules where the presence of the ions changes the geometry of these assemblies leading to energy transfer of quenching processes. Sensing can also be achieved with intrinsically nonfluorescent NPs as carriers, which have been conjugated with ion-responsive organic fluorophores.^1,2,10,11^ Regardless of the design, the active sensing element is typically located at the NP surface. The NP surface on the other hand also needs to provide colloidal stability, which is often achieved by using electrically charged surface coatings, so that NPs are stabilized by electrostatic repulsion.^12^ However, as known from basic physical chemistry, charged NP surfaces create a cloud of counterions in close vicinity of the surface (Debye–Hückel screening), which changes the local charge distribution near the NP surface, which, in turn, can be used for ion detection.^13^ In previous work, it was shown that attachment of pH-responsive fluorophores to the surface of negatively charged NPs leads to a localized increase in H^+^ concentration, i.e., a decrease of pH, at the NP surface due to electrostatic attraction (cf. Figure 1A,B; for the experimental data, see the Supporting Information (SI)).^1^ In the case of Cl^–^-responsive fluorophores attached to the surface of negatively charged NPs, a local decrease in the Cl^–^ concentration due to electrostatic repulsion was observed (cf. Figure 1C; for experimental data see the SI).^2^ This means that the ion concentration detected at the NP surface is different from the ion concentration in bulk. These effects have been shown to be distance-dependent, as expected from the Debye–Hückel theory. With increasing distance of an ion-responsive fluorophore to the NP surface (e.g., achieved by introducing molecular spacers of different length), the detected ion concentration approaches the bulk ion concentration.^1^ One may see such effects as experimental disadvantage: The readout of ion-responsive fluorophores in proximity to NP surfaces differs from that of free ion-responsive fluorophores, as the former detects the local ion concentration, whereas the latter detects the bulk ion concentration. Therefore, calibration of the readout is necessary to account for this difference.

On the other hand, the possibility to detect local concentrations, which depends on the properties of the ligand coating, can be seen as a distinct advantage. If low ion concentrations need to be detected, then a NP surface coating that electrostatically attracts ions could enhance the local ion concentration at the NP surface, enabling the detection of ion concentrations that would otherwise be too low for bulk detection with a free fluorophore. In other words, the ligand coating would facilitate an accumulation/enrichment effect. In cases in which high ion concentrations are beyond the response range of an ion-responsive fluorophore, a NP surface coating that electrostatically repels ions can be used to reduce the local ion concentration at the NP surface where the ion-responsive fluorophore is attached. This brings the ion concentration to a level that allows for a quantitative read-out. The NP surface coating thus may be used to tune the working range of ion-responsive fluorophores. There are two control parameters: the surface charge of the NP coating, which is mainly given by charged groups of the ligands, and the distance at which the ion-responsive fluorophores are immobilized with respect to the NP surface, which can be tuned by molecular spacers such as oligo(ethylene glycols) and poly(ethylene glycols) (PEG).

While enhancement/depletion effects of ions close to charged NP surfaces are governed by electrostatics, a quantitative description is not straightforward. One of the reasons is that there is no homogeneous NP surface for many NP surface coatings. Most coatings are not a simple monolayer of ligands, and molecular spacers are usually flexible to some extent, thinking, for example, about polymer brushes with a complex conformational behavior, which may include phase transitions and polymer conformational changes. Thus, there is no well-defined distance between the NP surface and an attached fluorophore but rather a radial distribution with a complex behavior. Theoretical calculations taking into account such effects can describe a general behavior that includes polymer conformational changes, and thus perfect match to experimental data requires fine-tuning of many experimental parameters (see the SI). Several other experimental data also demonstrate the effect. By using a Zn^2+^ responsive fluorophore close to a negatively charged surface, local accumulation of Zn^2+^ could be achieved and the working point of the Zn^2+^ sensor was shifted to lower Zn^2+^ concentrations (cf. Figure 1D; see the SI for the experimental data). Similar effects can also be achieved with positively charged ligand coatings. Cl^–^ ions are electrostatically attracted by positive surfaces; thus, the working point of fluorophores responsive to Cl^–^ in such environments shifts to lower Cl^–^ concentrations, as compared to the working point of the free fluorophores (cf. Figure 1E; see the SI for the experimental data). Moving the fluorophore away from the positively charged surface using molecular spacers negates this effect (cf. Figure 1E). This demonstrates the symmetry between negative and positive ions with respect to positively and negatively charged surfaces. These examples show that the working range of ion-responsive fluorophores attached to NPs can be adjusted by manipulating the ligand shell by changing the surface charge polarity and introducing molecular spacers. This allows for the detection of ion concentrations that are either lower or higher than the initial working range. The choice of the ligand shell thus allows for tuning the local environment and, thus, the functionality of the NPs. While this certainly may help to improve the working performance of ion-responsive fluorophores, there are also principal limitations. Electrostatic attraction/repulsion is not specific to the type of the ions, i.e., it has low selectivity, but it is only controlled by their charge. In a first approximation, a negatively charged surface would locally accumulate all positively charged ions and repel all negatively charged ions. There is a strong dependence on the valency of the ions: the electrostatic interaction is more pronounced for multivalent ions than for monovalent ions (see SI). However, in a first approximation, the local enrichment/depletion effect cannot distinguish between ions with the same sign of charge and valency. Moreover, the role of the ligand shell in maintaining the colloidal stability of NPs must also be considered. One approach is to use charged ligands that provide electrostatic repulsion between the NPs with the same charge. Nevertheless, the presence of ions can screen this charge and reduce the colloidal stability of NPs. Enrichment of small amounts of Na^+^ in acidic buffer (i.e. high H^+^ concentration) by integration of a Na^+^-responsive fluorophore into a negatively charged ligand shell of NPs thus would attract Na^+^ as well as H^+^ to the NP surface. While this could increase the local Na^+^ concentration to the point where it can be detected by the Na^+^-responsive fluorophore, there is a risk that the attracted H^+^ ions would completely screen the negative surface charge of the NPs, which would lead to their aggregation. The creation of a locally charged environment thus requires consideration of the effect of possible aggregation.

## Role of the Ligand Shell around NPs as a Local Nanoreactor

The experimental results in Figure 1 demonstrate that the concentration of dissolved molecules can differ significantly in the vicinity of NPs as compared with the bulk solution. This phenomenon can facilitate chemical reactions in the proximity of NP surfaces. This is distinct from the well-known fact that ligands around metal NPs can block the catalytic activity of the metal NP surface.^18−20^ Here, we refer only to the fact that certain molecular species can accumulate or be depleted close to NP surfaces. As reactions depend on the concentrations of the reactants, this in fact can convert the environment of NP surfaces to “nanoreactors”. This effect can be understood with the example of H^+^ accumulation close to negatively charged surfaces (cf. Figure 1A). Enzymatic reactions are, in general, pH dependent. Enzymes exhibit optimal activity within a specific pH range, and changes in pH can alter their three-dimensional structure, leading to a loss of function. Furthermore, H^+^ ions play a critical role as reactants or products in various enzymatic reactions, thereby affecting the local pH and altering the reaction equilibrium. In this way, the performance of enzymes immobilized at charged NP surfaces may be different from bulk. As can be deduced from Figure 1A,C (see the SI), the effect of ion accumulation/depletion reaches up to nanometer distances from charged NP surfaces, which is in the same range as the dimensions of enzymes. Sometimes in literature failure in the functionality of enzymes which are immobilized to charged NP surfaces is ascribed to steric hindrance, i.e., limited access of reactants/products, or to blocking of the active sites upon immobilization.^21^ While those effects certainly can play a role, the changes to the local environment (in particular, pH) also need to be considered. Those local effects can be relevant for charged hollow NPs such as polymer vesicles in particular, in which the interior is surrounded from all sides by charged surfaces.^22^ The effect of local environments is not limited to ions close to charged surfaces. Also polarity/hydrophobicity properties of the ligand shell around NPs may change local molecular concentrations.^23^ Organic fluorophores, for example, may accumulate in the hydrophobic parts of polymeric shells around NPs.^24^

As another example, appropriate nanoporous host structures provide a local environment with a variety of parameters, which can be adjusted to optimize local reactions. There is for example a series of reports about nanospace-confined chemical reactions in which NPs are grown.^25−27^ In general, confined molecules can fundamentally change their chemical and physical properties. Confinement effects are considered instrumental at various stages of life, and life continues to rely on layers of compartmentalization to maintain an out-of-equilibrium state and efficiently synthesize complex biomolecules under mild conditions. The principles governing reactivity under confinement are the same in abiological systems as they are in nature.^28^ Appropriate nanoporous materials provide confined spaces which can mimic the conditions for chemical reactions in cells and organelles, which are the places for the synthesis of essential building blocks, such as amino acids and sugars, including their oligomers and polymers. When the space surrounding molecules becomes restricted their reactivity and related behavior will be altered, and the role of water as solvent, including effects of local pH, dielectric constant, and ionic strength gradients, becomes dominant.^29^ Locally confined environments can, for example, be tailored to tune the catalytic activity of immobilized enzymes. While the porosity, which comprises the properties of surface area, pore diameter and volume, as well as pore dimensionality (1-, 2-, and 3D), determine the accessibility of the catalytic site, the surface chemistry defines the strength of interfacial interactions between the host and the enzyme.^3,30,31^ With its functional groups spatially distributed in an ordered fashion, the surface can act as a kind of “solid ligand”. Together both properties can increase the enzyme activity and its long-term stability because they can stabilize the active conformation and suppress the access of destabilizing agents. In addition, different enzymes can be immobilized in different host structures which can then be used in a modular enzyme cascade.^32^ Thus, in general, the local concentrations of different molecules can be different close to a NP surface (depending on the properties of the ligand shell) as compared to those in the bulk, which may change the equilibrium of reactions close to the surface.

Also in the here described examples of nanoreactors the ligand shell has low selectivity. Instead it may help to shift the local environment to conditions that favor certain reactions (for example, by modifying the local pH, which affects however many reactions and thus does not have high selectivity, or by limiting access to the reaction site by letting pass only molecules with a certain size, which again acts on all different types of molecules with the appropriate size and thus does not have high selectivity). Again, the key is to use the ligand shell as a low selectivity filter/modulator that influences the actual reaction.

## Fishing for Proteins

While the effect of tuning the working point of ion-responsive fluorophores by different ligand coatings of NPs is barely discussed in literature, a similar conceptual phenomenon, the ligand-dependent formation of a protein corona around a NP surface has been heavily investigated.^33^ Due to the high number of recent reviews about this topic, in this Account the concept is only briefly described.^34−36^ Proteins adsorb to the surface of NPs.^37^ Adsorption depends on the local surface properties dictated by the ligand shell, such as surface charge and polarity distributions.^38,39^ Depending on the ligand shell, different proteins can adsorb to the surface of NPs.^40,41^ Certain proteins preferentially bind to the NP surface, leading to a higher local concentration at the surface compared to the bulk.^42^ In this way, it is possible to “fish out” certain proteins with NPs. This has particular importance for molecular diagnostics. Proteins present in low abundance in the blood can be enriched at the surface of NPs with tailored ligand coatings, thereby rendering them more accessible for diagnostic purposes. There are two distinct strategies. In case when there is only one protein of interest whose presence is to be detected, then antibodies against this protein would be added as ligands to the NP surface. The binding thus would be highly selective for the target protein. However, for many diseases there may be no particular “target” protein. Instead, several different proteins may be up- or downregulated.^43,44^ Some of those proteins may be low-abundance and thus hard to detect by standard proteomics approaches (which are often based on mass spectrometry). In general, the number of protein types that can be detected by mass spectrometry from blood is limited and low abundance proteins are missed. Here NPs can help. In this case, NPs with different ligand shells are added to blood; depending on the particular ligand shell, different types of proteins will be found on the NP surface. Applying mass spectrometry based proteomics on a library of NPs which have been exposed to blood instead of directly detecting the proteins from blood yielded a higher number of detected types of proteins.^45−47^ In this way, by enriching proteins on different NP surfaces, the up- or downregulation of more types of proteins could be detected. Again, this makes use of low selectivity in binding. Different ligands favor other proteins to adsorb onto the NPs. The trick hereby is to make a minimum library of different ligand coatings, for which in total most proteins will adsorb to the NPs. “Fishing” of whole classes of proteins by ligands with low selectivity thus can be favorable compared to only extracting one type of protein with highly selective ligands.

## Conclusions

The ligand shell surrounding dispersed NPs may act as an “attractor” or “repellant” for certain molecules in the solvent. The local concentrations of those molecules close to the NP surfaces can be significantly different from their bulk concentrations. Those effects can be viewed as positive or negative. On the negative side, reactions occurring at the NP surface may differ from those in bulk, leading to failed detection of the same analyte at the NP surface. Understanding the concept of the local environment is, therefore, crucial to avoid such failures. On the positive side, the ligand shell can be tailored to locally accumulate molecules to be detected or extracted. For example, the electrostatic attraction of ions to charged surfaces can shift the working point of their detection. With more elaborate ligand coatings, adsorption of a maximum number of different types of proteins from complex matrices like body fluids may be achieved. The ligand shell can also be used as a local nanoreactor, allowing for reaction conditions different from bulk, thus enhancing reaction rates or even modulating reactions to take place exclusively at the NP surface. It might also be used to tune reactions via the properties of the reactants. In combination with additional surface-distance-dependent effects such as local heating and field enhancement in plasmonic photocatalysis, highly selective and active catalysts are conceivable.

While in general it could be argued that ligand shells should possess high selectivity for the binding of molecules of interest, in this Account, a point is made about the virtues of ligands with low binding selectivity. While less selective, the effect of such ligand shells may act over longer distances and also instead of targeting one type of molecule may target a whole class of molecules. There is a conceptual virtue of such low selectivity surfaces as filters and preconcentrators of molecules close to the surface of NPs.

## Supporting Information Available

Detailed protocol for the synthesis and characterization of the NPs with ion-responsive fluorophores, additional results for ion sensing with ion-responsive fluorophores, and theoretical analysis of the ion distribution near charged NP surfaces (PDF)
