# Supplementary material for: Local Environments Created by the Ligand Coating of Nanoparticles and Their Implications for Sensing and Surface Reactions
Source: Acc Chem Res. 2023 Aug 22;56(17):2278–85. doi: 10.1021/acs.accounts.3c00139 (PMC10552541; doi:10.1021/acs.accounts.3c00139)
Supplement: Supplementary file 1 — ar3c00139_si_001.pdf [file ar3c00139_si_001.pdf]

Local Environments Created by the Ligand Coating of Nanoparticles and Their Implications for Sensing and Surface Reactions

Florian Schulz[1], Jonas Hühn[2], Marco Werner[3], Dominik Hühn[2], Julia Kvelstad[4], Ulrich Koert[4], Nicole Wutke [5], Markus Klapper[5], Michael Fröba[6], Vladimir Baulin[7*], Wolfgang J. Parak[1*]

[1]Fachbereich Physik, Universität Hamburg, 22607 Hamburg, Germany
[2]Fachbereich Physik, Philipps Universität Marburg, 35037 Marburg, Germany
[3]Leibniz-Institut fur Polymerforschung Dresden e.V., 01069 Dresden, Germany
[4]Fachbereich Chemie, Philipps Universität Marburg, 35043 Marburg, Germany
[5]Max Planck Institute für Polymerforschung, 55128 Mainz, Germany
[6]Fachbereich Chemie, Universität Hamburg, 20146 Hamburg, Germany
[7]Departament Quimica Fisica i Inorganica, Universitat Rovira i Virgili, 43007 Tarragona, Spain
[*]corresponding authors: va.baulin@gmail.com, wolfgang.parak@uni-hamburg.de

SUPPORTING INFORMATION

1 Synthesis, purification and characterization of ion responsive NPs
1.1 Synthesis of dodecanethiol stabilized gold nanoparticles (Au NPs)
1.2 Synthesis of ion reponsive dyes
1.3 Synthesis of amphiphilic polymers equipped with ion reponsive dyes
1.4 Coating of hydrophobic Au NPs with amphiphilic polymers
1.5 Purification of polymer coated Au NPs
1.6 UV/Vis absorption spectroscopy

2 Examples for ion-sensing
2.1 Measurement set-up
2.2 H^+-sensing with SNARF attached to negatively charged polymer with spacers
2.3 H^+-sensing with OG attached to negatively charged polymer
2.4 Cl^--sensing with MAQE attached to negatively charged polymer with spacers
2.5 Zn^{2+}-sensing with AMQB attached to negatively charged polymer
2.6 Cl^--sensing with MAQE attached to positively charged polymer with spacers
2.7 Summary of sensing concept

3 Ion-response simulations for negatively charged surfaces

4 References

1 Synthesis, purification and characterization of ion responsive NPs

1.1 Synthesis of dodecanethiol stabilized gold nanoparticles (Au NPs)

Hydrophobic dodecanethiol stabilized gold nanoparticles (Au NPs) were synthesized according to standard procedures based on the method by Brust *et al.*[1-3] Two solutions were made, one with 300 mg of $HAuCl_4$ (0.88 mmol, 1 eq, Alpha Aesar, # 42803) dissolved in 20 mL Milli-Q water and one with 2.17 g of tetraoctylammonium bromide (TOAB, 3.97 mmol, 4.5 eq, Sigma-Aldrich, # 294136) that was dissolved in 80 mL of toluene (Fluka 89682). These solutions were mixed in a separatory funnel and gently shaken for 5 min. During mixing the initial $Au^{III}Cl_4^-$ ions were gradually transferred from the yellow aqueous solution into the organic toluene phase, by forming tetraoctylammonium tetrachloroaurate ion pairs ($N(C_8H_{17})_4^+AuCl_4^-$). This process was indicated by a color change of both phases, where the toluene phase (tol) changes from transparent to deep orange and the aqueous phase (aq) become transparent. Showing the effective $AuCl_4^-$-ion transfer to the organic phase, the aqueous phase became transparent and was drained from the mixture. Another solution of 334 mg of $NaBH_4$ (8.82 mmol, 10 eq, Sigma-Aldrich, # 452882) dissolved in 20 mL Milli-Q water was prepared. After some minutes of stirring the aqueous sodium borohydride solution, small bubbles, due to hydrogen formation were observed. As hydrolysis of BH_4^- would decompose the needed reducing agent, the solution should always be prepared freshly and used immediately. The solution of sodium borohydride was added dropwise into a round flask containing the stirring organic layer. A color change from red to violet was observed according to the reduction of $AuCl_4$ to Au^0 and the formation of Au clusters. To make sure all remaining Au^{III} ions were reduced, the solution was kept stirring at room temperature (RT) for 1 h. After stirring for one hour at room temperature (RT), the mixture was transferred into a separatory funnel. The aqueous phase was discarded and 25 mL of 10 mM HCl were added. The solution was gently shaken for one minute to remove the excess of HCl from the organic layer to wash out any remaining salts or precursors in the organic phase. After the aqueous phase was discarded, 25 mL of 10 mM NaOH were added and the solution was again shaken gently for one minute, removing any excess of acidic residues. The aqueous layer was drained and the organic phase was washed 3 times with 25 mL Milli-Q water to remove any salts or aqueous impurities. The deep red organic solution was transferred into a round flask and stirred over night at RT. This step is recommended and will lead to thermodynamically stable Au NPs with a narrow size distribution, mediated by Ostwald ripening. To perform a ligand exchange between TOAB molecules by dodecanethiol (DDT), 10 mL of 1-dodecanethiol (41.7 mmol, 47.5 eq, Sigma-Aldrich, # 471364) were added while the flask with the organic solution was immersed into a preheated oil bath at 65°C. The solution was stirred for 3 hours under reflux. Now the solution was split and transferred into several 40 mL glass vials to centrifuge them for 5 minutes at 2000 rpm. The supernatant was collected in 40 mL glass vials (10 mL each) and the precipitates containing bigger aggregates at the bottom were discarded. The glass vials with the supernatants containing the Au NPs were filled up with anhydrous methanol (Sigma-Aldrich, # 322415) and centrifuged for 5 minutes at 2000 rpm, what led to precipitation of Au NPs. The transparent supernatant, containing unwanted remains, was discarded and the solid Au NP residues were dissolved in the minimum possible amount of anhydrous chloroform (Sigma-Aldrich, # 372978). The obtained Au NPs were now ready for coating with amphiphilic polymers.

The synthesized hydrophobic dodecanethiol stabilized Au NPs were characterized by transmission electron microscopy (TEM) and UV/Vis absorption spectroscopy (Figure S1). From the TEM images the

diameter of 1669 Au NPs was measured using the public domain software ImageJ with the "Particle Size Analyzer" (PSA) macro freely available at http://code.google.com/p/psa-macro/. The result was fitted with a Gaussian providing a mean value for the Au NP core diameter d_c of 4.0 ± 1.3 nm. The effective diameter d_{eff}, which is the sum of the core diameter and the surfactant shell was then estimated to be 6.0 nm with a length of the alkyl chains of approximately 1 nm.[4-6] The effective diameter $d_{eff} = d_c + 2 \cdot l_{surfactant}$ was used to calculate the required amount of polymer for the coating process (*cf.* § III). The absorbance profile of hydrophobic Au NPs in chloroform showed a surface plasmon resonance (SPR) at approximately 518 nm.

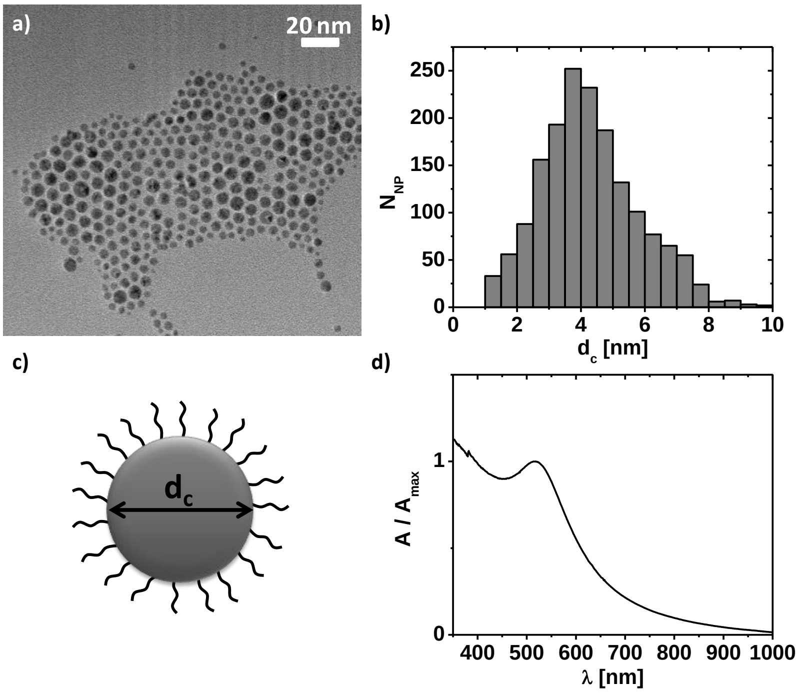

Figure S1: a) TEM image of dodecanethiol stabilized Au NP cores. The dodecanethiol shell does not provide contrast.[3] b) The core diameter d_c of 1669 NPs was evaluated with ImageJ and plotted in a histogram in which the number of counted NPs (N_{NP}) of certain diameter was counted with a bin size of 0.5 nm. A Gaussian fit delivered a mean NP diameter of d_c = 4.0 ± 1.3 nm (mean value ± standard deviation). c) Schematic drawing of the dodecanethiol capped Au NPs of core diameter d_c. d) Extinction spectrum of dodecanethiol stabilized Au NPs in chloroform normalized to the absorbance at the SPR peak A_{max} located at 518 nm.

The concentration c_{NP} of the Au NPs was determined using the Lambert-Beer law (Equation S1).[3]

$$c_{\mathrm{NP}} = \frac{\mathrm{A}}{\varepsilon \mathrm{l}} \qquad \text{Equation S1}$$

Here A is the absolute absorbance at the SPR peak, ε is the molar extinction coefficient and l is the cuvette length (1 cm). ε was calculated to be $4.92 \cdot 10^6$ $M^{-1}cm^{-1}$ for Au NPs of 4.0 nm core diameter from the literature.[7]

1.2 Synthesis of ion reponsive dyes

1.2.1 Synthesis of amino-MQAE
1.2.2 Synthesis of AMQB

1.2.1 Synthesis of amino-MQAE

The chloride reponsive dye 2-(2-(6-methoxyquinoliniumchloride)ethoxy)-ethanamine-hydrochloride (amino-MQAE) was used and synthesized as described in an earlier study.[8]

1.2.2 Synthesis of AMQB

The Zn^{2+} reponsive fluorophore 4-aminomethyl-*N*-(6-methoxy-quinolin-8-yl) benzenesulfonamide (AMQB), was synthesized according to the literature.[9]

1.3 Synthesis of amphiphilic polymers equipped with ion reponsive dyes

1.3.1 Synthesis of negatively charged polymer with PEG spacers with Cl^- responsive dye (MQAE) and CVP as reference dye for ratiometric read-out
1.3.2 Synthesis of negatively charged polymer with Zn^{2+} responsive dye (AMQB) and CVP as reference dye for ratiometric read-out
1.3.3 Synthesis of positively charged polymer with Cl^- responsive dye (MQAE)
1.3.3 Synthesis of positively charged polymer with Cl^- responsive dye (MQAE)
1.3.4 Synthesis of positively charged polymer with PEG spacers with Cl^- responsive dye (MQAE) and CVP as reference dye for ratiometric read-out

1.3.1 Synthesis of negatively charged polymer with PEG spacers with Cl^- responsive dye (MQAE) and CVP as reference dye for ratiometric read-out

A negatively charged amphiphilic polymer was functionalized with amino MQAE or amino-PEG-modified amino MQAE following previously published protocols.[8] 1 eq maleic acid anhydride rings (monomers of poly(isobutylene-*alt*-maleic anhydride) PMA, M_W = 6 kDa) were stirred with 0.75 eq dodecylamine, 0.02 eq amino MQAE, 0.02 eq cresyl violet perchlorate (CVP), 0.1 eq 4-(dimethylamino)pyridine (DMAP), and 0.12 eq triethylamine (TEA) for 18 h at 80 °C under reflux in tetrahydrofuran (THF). After the conjugation, in which the amino groups of MQAE and CVP formed amide bonds with the carboxylic groups of the polymer, the solvent was evaporated and the dry film dissolved in chloroform.

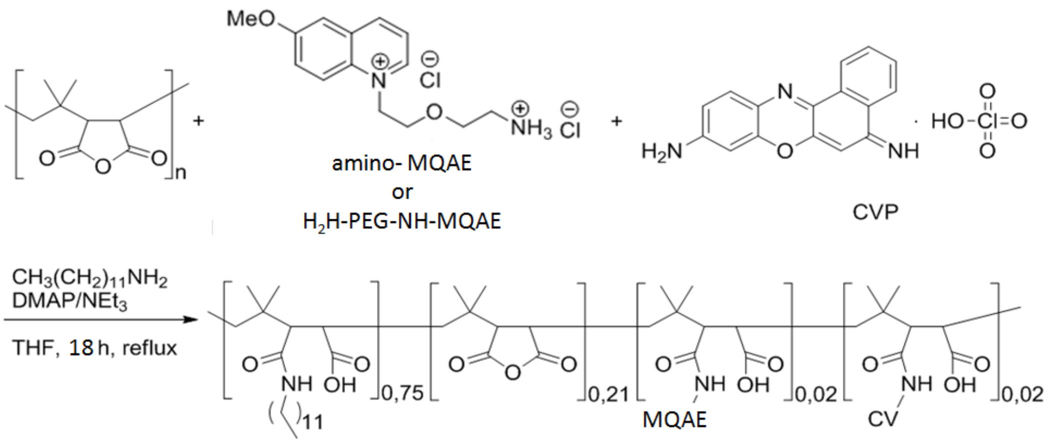

Figure S2: Reaction scheme for the functionalization of a negatively charged amphiphilic polymer with amino MQAE or amino-PEG-modified amino MQAE.[8]

1.3.2 Synthesis of negatively charged polymer with Zn^{2+} responsive dye (AMQB) and CVP as reference dye for ratiometric read-out

The amphiphilic polymer based on poly(isobutylene-*alt*-maleic anhydride) (PMA) was modified with hydrophobic sides chains as well as with two dye molecules. Compount 11 of Teolato *et al.*[9], a Zn^{2+} reponsive dye, in the following denoted as 4-aminomethyl-*N*-(6-methoxy-quinolin-8-yl)-benzenesulfonamide (AMQB), and cresyl violet (CV; cresyl violet perchlorate = CVP) as intramolecular reference dye.

1.54 g (10 mmol (of PMA monomers), 1.0 eq) of poly(isobutylene-*alt*-maleic anhydride) (M_W ~6000 g/mol of one polymer chain, with the mass of one monomer unit of M_{PMA} = 154 g/mol, thus one polymer chain carries approximately 39 unites) (Sigma-Aldrich, no. 531278) were filled in a 250 mL round bottom flask and mixed with 1.35 g (7.5 mmol, 0.75 eq) dodecylamine (DDA, ≥ 98%, Fluka, no. 44170) dissolved in 50 mL anhydrous tetrahydrofuran (THF, ≥99.9%, Aldrich, no. 186562). Also 68.7 mg (0.2 mmol, 0.02 eq) AMQB, 72.3 mg (0.2 mmol, 0.02 eq) cresyl violet perchlorate (Sigma Aldrich, no. 255246), 122 mg (1 mmol, 0.1 eq) 4-(dimethylamino)pyridine (DMAP; Sigma Aldrich, no. 107700) and 167 µL (1.2 mmol, 0.12 eq) triethylamine (TEA; Sigma Aldrich, no. 471283) were added to the reaction mixture. The mixture was then sonicated for ca. 1 min. The solution was heated up to 80°C (oil bath) and after 10 min the solution turned clear which indicated the complete dissolution of PMA, AMQB and CVP. The solution then was stirred under reflux over night. Afterwards the sample was cooled down to RT and the solvent was removed under reduced pressure. After evaporation of the solvent, the dry film was dissolved in 20 mL anhydrous chloroform (Sigma, no. 288306), leading to a 0.5 M solution respective PMA monomer units. By this 75% of maleic anhydride rings reacted with dodecylamine, 2% with AMQB, and another 2% with CV, leaving 21% of anhydride rings unreacted. During phase transfer of the polymer coated Au NPs from organic to aqueous solution (*cf.* chapter 1.4), the free and deprotonated carboxyl groups provide a negative surface charge of the Au NPs and their colloidal stability. This polymer solution was ready to use for coating hydrophobic Au NPs without further purification (*cf.* chapter 1.4).

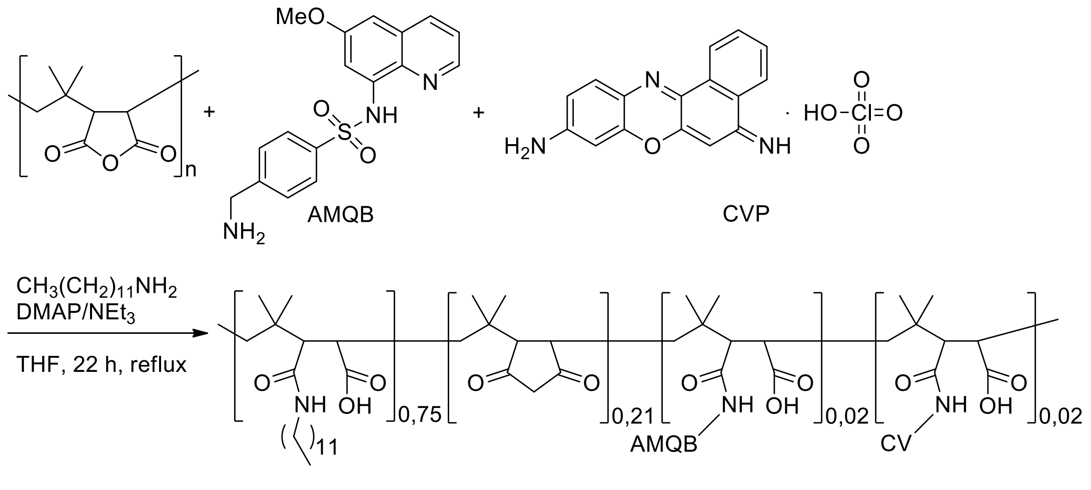

Figure S3: Reaction scheme for functionalizing a negatively charged amphiphilic polymer with AMQB. After the conjugation, in which the amino groups of AMQB and CVP form amide bonds with the carboxylic groups of the polymer, the solvent is evaporated and the dry film dissolved in chloroform. The polymer is used for coating without further purification.

1.3.3 Synthesis of positively charged polymer with Cl^- responsive dye (MQAE)

An amphiphilic polymer, which comprises hydrophilic (x), hydrophobic (y), and functional (z) monomers was equipped with the chloride reponsive dye amino-MQAE (chapter 1.2.1) *via* a crosslinker. As the functional group of the polymer is a thiol (SH) group and the dye provides a primary amino group the heterofunctional crosslinker sulfosuccinimidyl 4-(*N*-maleimidomethyl)cyclohexane-1-carboxylate (sulfo-SMCC, Thermo Scientific, # 22322) is suited for the conjugation reaction. The maleimide group forms a stable carbon-sulfur bond with the functional polymer whereas the NHS-ester reacts with the amino function of the dye molecule to form an amide bond.

The positively charged amphiphilic terpolymer, based on PTMAEMA-stat-PLMA (PTMAEMA = poly(*N*,*N*,*N*-trimethylammonium-2-ethyl methacrylate iodide), PLMA = poly(lauryl methacrylate)) is synthesized as in a former protocol.[2] The terpolymer consist of 3 different monomeric unites with a polydispersity index (PDI) of 1.6. A positively charged component based on a quaternary ammonium cation (x = 3%), a hydrophobic unit based on an aliphatic side chain (y = 48.5%) and a thiol group (z = 48.5%).

The polymer was functionalized using sulfosuccinimidyl-4-(*N*-maleimidomethyl)-cyclohexane-1-carboxylate (sulfo-SMCC) as crosslinker and the Cl^- reponsive dye 2-[2-(6-methoxyquinolinium chloride)ethoxy]-ethanamine hydrochloride (amino-MQAE, synthesized as reported previously (chapter 1.2.1).[8]) The crosslinker as well as the dye were added surplus in a “one-pot-reaction” to ensure an efficient functionalization. Although sulfo-SMCC is subject to hydrolysis, water was chosen as solvent following the instructions of the crosslinker manual.

To this end, 50 mg (1.0 eq, 2.31 µmol of SH groups) of the polymer were stirred with 10 mg (10 eq, 23.1 µmol) sulfo-SMCC (Thermo Fisher, no. 22322), and 73.1 mg (100 eq, 231 µmol) amino-MQAE for 18 h at room temperature (RT) in 1 mL milliQ water. After the conjugation, in which the maleimide

group reacted with the SH group of the polymer and the *N*-hydroxysuccinimide (NHS) residue was released under an amide bond formation with the amino function of the dye, the polymer was purified from unreacted crosslinker and dye molecules *via* centrifugation. The whole sample was transferred into a centrifuge filter (Amicon Ultra-15, NMWL = 3 kDa, Merck Millipore, # UFC900324) and washed with fresh Milli-Q water until the flow through didn't show any more fluorescence. Then, the water was evaporated under reduced pressure and a solid film remained. Some loss of material was mainly caused by the washing procedure as parts of the polymer might stick to the filter membrane. At this point the exact conjugation efficiency was not known and a yield of 50% was assumed for further calculations. This assumption is reasonable as the molecular weight of the conjugate is mainly determined by the molecular weight of the polymer. After transfer of the polymer to chloroform, with a concentration of 0.05 M of hydrophobic side chains, it was readily usable for the coating of Au NPs (see chapter 1.4). The attachment of CV as reference dye was not possible in the present case due to solubility reasons, but the issue of normalization was overcome as will be described in chapter 2.6.

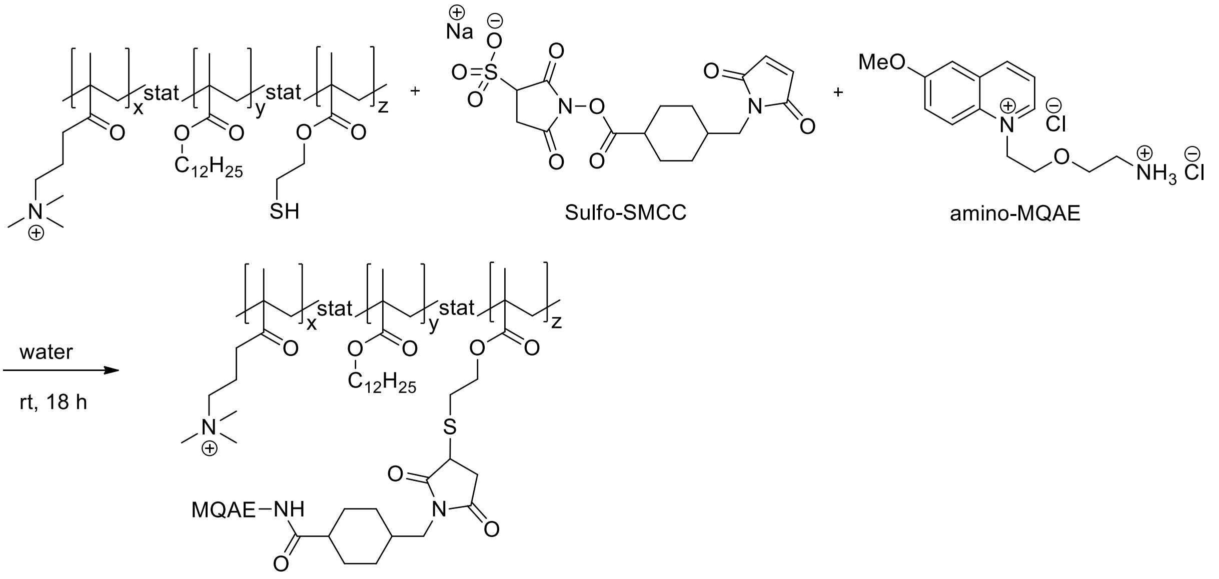

Figure S4: Reaction scheme for the functionalization of a positively charged amphiphilic terpolymer with MQAE using the crosslinker molecule sulfo-SMCC. By working in excess of crosslinker and dye, most thiol groups should be reacted and functionalized under the given parameters.

1.3.4 Synthesis of positively charged polymer with PEG spacers with Cl^- responsive dye (MQAE) and CVP as reference dye for ratiometric read-out

In a second approach to link MQAE to a positively charged polymer (cf. chapter 1.3.3) another crosslinker, namely succinimidyl-([*N*-maleimidopropionamido]-2-ethylene glycol ester ($SM(PEG)_2$, Thermo Scientific, # 22102), was used for the conjugation of the dye to the polymer. The motivation was to determine if a longer spacer arm length has an impact on the sensitivity. However, the functional groups of the crosslinker are the same as compared to sulfo-SMCC, so that the conjugation procedure was carried out with only slight differences see (Figure S5). Additionally, CVA was attached as reference dye.

To this end, 50 mg (1.0 eq, 2.31 µmol of SH groups) of the polymer were stirred with 9.83 mg (10 eq, 23.1 µmol) SM(PEG)$_2$ (Thermo Fisher, no. 22102), 66.4 mg (90 eq, 209.7 µmol) amino-MQAE and 8.36 mg (10 eq, 23,1 µmol) CVP (Sigma Aldrich, no. 255246) for 18 h at RT in 1 mL phosphate buffered saline (PBS) / ethanol (1:2). All unwanted remains, like unreacted crosslinker or dye molecules, were extracted by centrifugation (*cf.* chapter 1.3.3). The functionalized polymer was transferred to chloroform, with a concentration of 0.05 M of hydrophobic side chains, and was readily usable for the coating of Au NPs.

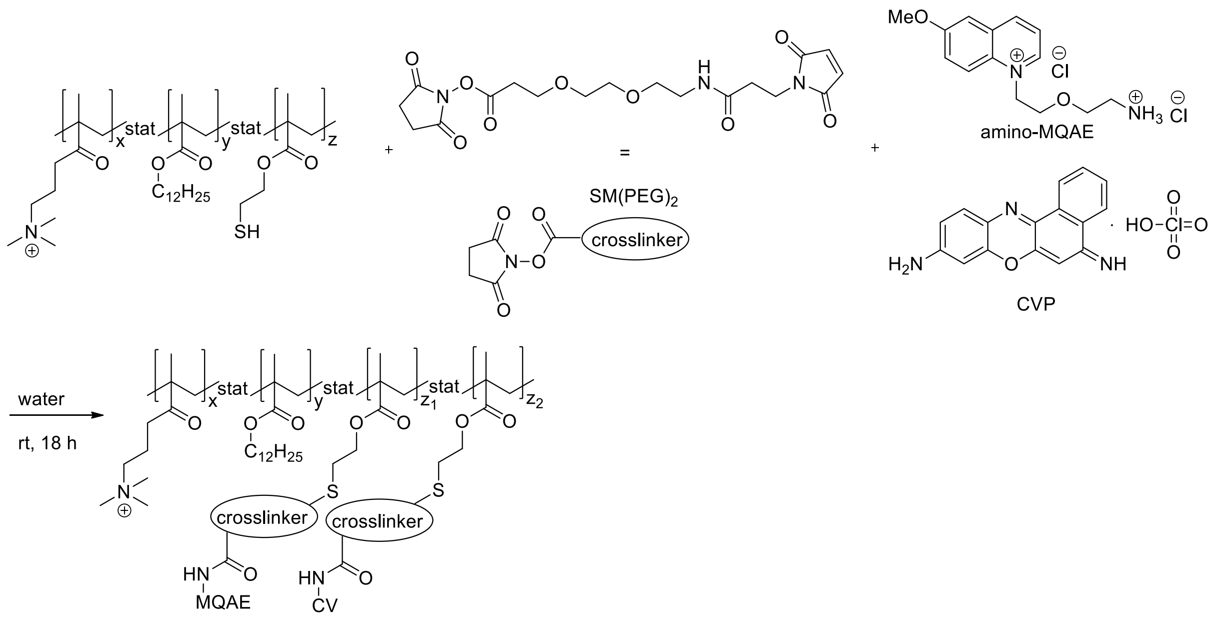

Figure S5: Reaction scheme for the functionalization of a positively charged amphiphilic polymer with MQAE using the crosslinker molecule SM(PEG)$_2$. CVP serves as intrinsic fluorescent normalization probe (reference fluorphore). The remaining procedure is the same as compared to the conjugate with sulfo-SMCC (Figure S4).

1.4 Coating of hydrophobic Au NPs with amphiphilic polymers

1.4.1 Sketch and description of the general NP geometry

1.4.2 Coating with negatively charged amphiphilic polymers

1.4.3 Coating with positively charged amphiphilic polymers

1.4.1 Sketch and description of the general NP geometry

Small analyte molecules, such as in particular ions can be detected with analyte-reponsive fluorophores (F). In general, upon binding of the analyte to the analyte-reponsive fluorophore the fluorescence emission of the fluorophore changes. In ratiometric measurements, a second (reference) fluorophore (R) can be added, whose emission intensity does not depend on the presence of the analyte, see Figure S5. Thus, the ratio of the emission of the analyte-reponsive fluorophore and the emission of the reference fluorophore depends on the concentration of the analyte.

Linkage of the fluorophores to the surface of colloidal nanoparticles (NPs) changes the response of the fluorophores to the presence of analyte molecules.[8,10,11] This effect will be discussed in chapter 2 for ion-reponsive fluorophores in which ions are the analytes to be detected. The NPs are coated with an organic coating that bears charged moieties (C), see Figure S6. These charged moieties stabilize the NPs in aqueous solution by electrostatic repulsion.[12] However, they also attract counter ions from solution, an effect which is known as Debye-Hückel screening. In case the NP surface is charged negatively, positively charged ions from the surrounding solution will be locally attracted, whereas negatively charged ones will be locally repelled. In the case of positively charged NPs the effect is reverse: local attraction and repulsion of negatively and positively charged ions, respectively. In this way, the charged NP surface creates a local environment close to the NP surface in which the concentrations of ions are different from bulk concentrations.[13] Screening is in general non-specific to the chemical nature of the ions and in first order depends only on their charge.

The ion-responsive fluorophores (F) can be linked to the NP surface *via* molecular spacers (S) of variable length (the "shortest" length hereby corresponds to no spacer), see Figure S6. Thus, in case the ion-reponsive fluorophores are linked directly to the NP surface (none or very short molecular spacers used for attachment), they will sense the local ion concentration close to the NP surface. On the other hand, in case the ion-reponsive fluorophores are far away from the NP surface (long molecular spacers used for their attachment), they will sense bulk ion concentrations.[8,10,11] In other words, attachment of ion-reponsive fluorophores to the surface of charged NPs changes their response to the presence of the ions to be detected, whereby the response depends on the distance of the fluorophores to the NP surface. The reference fluorophores (R) can be directly linked to the NP surface or also *via* spacers, whereby their distance to the NP surface is of no importance.

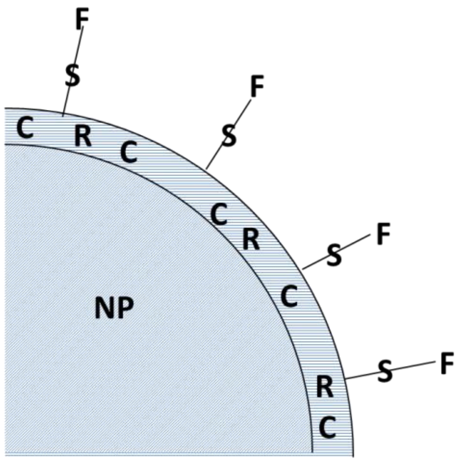

Figure S6: A nanoparticle (NP) core is surrounded by a (in general semi-permeable) organic coating which comprises charged groups (C) and reference fluorophores (R). Analyte-reponsive fluorophores (F) are linked *via* molecular spacers (S) to the surface of the NP.

In chapter 1.1 the synthesis of the Au NPs has been described and in chapter 1.3 the synthesis of amphiphilic polymers with different integrated ion-responsive fluorophores. In Figure S7 an overview about the different fluorophore-modified polymers as used in this study is given.

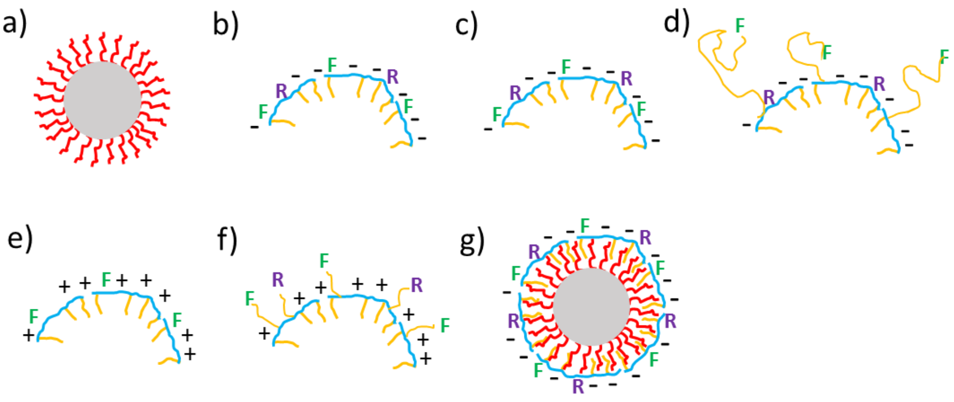

Figure S7: Sketch of the geometry of the used NPs and their coatings. a) Au NPs (core drawn in grey) with dodecanethiol capping (red, *cf.* chapter 1.1). b) Negatively charged amphiphilic polymer, with hydrophobic side chains (orange) and hydrophilic backbone (blue). CV as reference dye (R) and AMQB (F) as Zn^{2+} reponsive fluorophores are directly integrated in the polymer (*cf.* chapter 1.3.2). c) Same geometry as b), but with the Cl^{-}-reponsive dye MQAE (F) (*cf.* chapter 1.3.1). d) MQAE (F) is linked via PEG-spacers (orange) to the surface of the negatively charged polymer (*cf.* chapter 1.3.1). e) Positively charged amphiphilic polymer, with MQAE (F) linked to the polymer shell (*cf.* chapter 1.3.3). f) MQAE (F) and CV (R) are linked to the positively charged polymer via PEG spacers (orange) (*cf.* chapter 1.3.4). g) Once the amphiphilic polymer (here as example the one from b)) is wrapped around the hydrophobic Au NPs (as shown in a)), the NPs are rendered water-soluble (*cf.* chapter 1.4.2 and chapter 1.4.3).

1.4.2 Coating with negatively charged amphiphilic polymers

The polymer coatings of hydrophobic Au NPs described herein were already discussed in detail in earlier publications.[5,8] However, a brief description will be given in the following. The amphiphilic, dye functionalized polymer as synthesized in chapter 1.3 served for the transfer of hydrophobic, dodecanethiol stabilized Au NPs (chapter 1.1) from an organic to an aqueous environment. The polymer that was used for the coating contains hydrophobic dodecylamine side chains which represent one part of the amphiphilic nature of the polymer. Upon mixing of hydrophobic Au NPs and amphiphilic polymer the alkyl chains of each partner intercalate so that a shell is formed around the particle. By evaporation of the solvent ($CHCl_3$) and dissolution in an aqueous solvent the polymer shell tightens due to strong hydrophobic interaction. Additionally, the anhydrides in the polymer backbone hydrolyze, thus generating a net negative charge *via* the formation of carboxylic acid groups. This leads to sufficient electrostatic repulsion between individual NPs to form a colloidally stable dispersion.

According to the size evaluation of the hydrophobic Au NPs in chapter 1.1, which led to a d_{eff} of 6.0 nm, the volume V_p of polymer solution (c_p = 0.05 M of hydrophobic groups) needed for the coating of the NPs was estimated as follows (Equation S2):[3]

$$V_p = \frac{n_p}{c_p} = \frac{A \cdot R_p}{N_A \cdot c_p} = \frac{R_p \cdot \pi \cdot c_{NP} \cdot V \cdot d_{eff}^2}{c_p} \qquad \text{Equation S2}$$

Herein n_p is the molar quantity of hydrophobic side chains in the polymer, A is the surface area of all present Au NPs (according to d_{eff}) in nm^2, R_p is the number of hydrophobic side chains (of the polymer) added per nm^2, N_A is the Avogadro constant, c_{NP} is the concentration of Au NPs (for the determination see chapter 1.1 and Hühn *et al.*[3]), and V is the volume of Au NP solution. In most of the cases, the factor R_p was chosen as 50 nm^{-2} but partly it was also increased to 100 nm^{-2} (higher excess of polymer) in case the coating process didn't work out perfectly. However, it is desirable to keep R_p as small as possible to minimize the amount of excess polymer and thus to increase the purity of the obtained Au NPs solution.

To perform the coating, Au NPs suspended in chloroform were mixed with a certain amount of polymer (*cf.* Equation S2) in a round bottom flask and connected to a rotary evaporator. The mixture was stirred at 40 °C for about 5 min under atmospheric pressure. Then the solvent was slowly removed under reduced pressure until complete dryness. A few mL chloroform were added and the Au NPs were resuspended. Again, the mixture was stirred for about 5 min at 40 °C and subsequently the solvent was slowly evaporated. This process was performed 3 times in total. After the last evaporation step the dried Au NPs were dissolved in aqueous buffer. In case of the negatively charged polymer, sodium borate buffered saline at pH 12 (SBB 12) was used. By this, all remaining anhydride rings were hydrolyzed and the carboxylic groups deprotonated, generating a negative surface charge of the NPs. Once the Au NPs were dissolved in the aqueous buffer, ultrasonication was optionally used to obtain a higher yield of Au NPs. The suspension was then pushed through a 0.22 µm syringe filter to get rid of bigger Au NP agglomerates. The Au NPs were cleaned in 15 mL centrifugation filters (pore size 100 kDa) and concentrated to be separated from excessive polymer fragments by gel electrophoresis (*cf.* chapter 1.5.1).[3,8,14]

1.4.3 Coating with positively charged amphiphilic polymers

The procedure of polymer coating to obtain positively charged Au NPs was similar to the one for negatively charged Au NPs (chapter 1.4.2). The slight differences are mentioned in the following. An Au NPs solution was added to the as synthesized polymer (see chapter 1.3) with the same calculation of V_p (Equation S2) according to the amount of present hydrophobic side chains. The corresponding amount of Au NPs was added, considering a R_p factor of 50 nm^{-2}. After the third evaporation step for removal of the organic solvent approximately 1 mL of 0.1 M NaCl at pH = 3.3 (adjusted with HCl) was added for the transfer in water. Directly after the transfer to an aqueous solvent all samples were purified from unavoidable agglomerates through a 0.22 µm syringe filter. The coating process did not work out with the polymer before it was functionalized with the dye. Possible reasons for this might be an interaction of the thiol groups present in the polymer backbone with the Au cores or the formation of disulfide bonds in-between polymer strands. However, when the SH groups were vented before the coating process, the latter worked, though not as efficient as with the negatively charged counterpart.

1.5 Purification of polymer coated Au NPs

1.5.1 Purification of negatively charged Au NPs

1.5.2 Purification of positively charged Au NPs

1.5.1 Purification of negatively charged Au NPs

The purification *via* gel electrophoresis was only feasible with negatively charged Au NPs as positively charged Au NPs did not migrate in the used agarose gel matrix. Gel electrophoresis allows for both, the purification of the Au NPs from excess polymer or other impurities and a colloidal characterization. [15] The narrowness of the particle band gives information about the polydispersity and the traveled distance about the charge per size relation.

The solution to be purified was concentrated in a centrifuge filter (3000 rpm, molecular weight cut-off (MWCO) = 100 kDa, Satorius Stedim, # VS2042) to a final NP concentration of approximately c_{NP} = 1 µM and 20 vol.% of gel loading buffer (GLB, 25 mL glycerol, 35 mL 0.5× tris-borate-EDTA-buffer (TBE, Sigma-Aldrich # T3913), 130 mg Orange G (Sigma-Aldrich, # 861286) were added. The mixture was carefully pipetted into the well of a 2 wt% agarose gel (UltraPure™ Agarose, Invitrogen, # 16500500) in TBE buffer that was placed in an electrophoresis device, filled with 0.5× TBE buffer and run in general for 60 min with an electric field of 10 V/cm. Figure S8 shows an exemplary batch of Au NPs, coated with the negatively charged polymer functionalized with AMQB and CV.

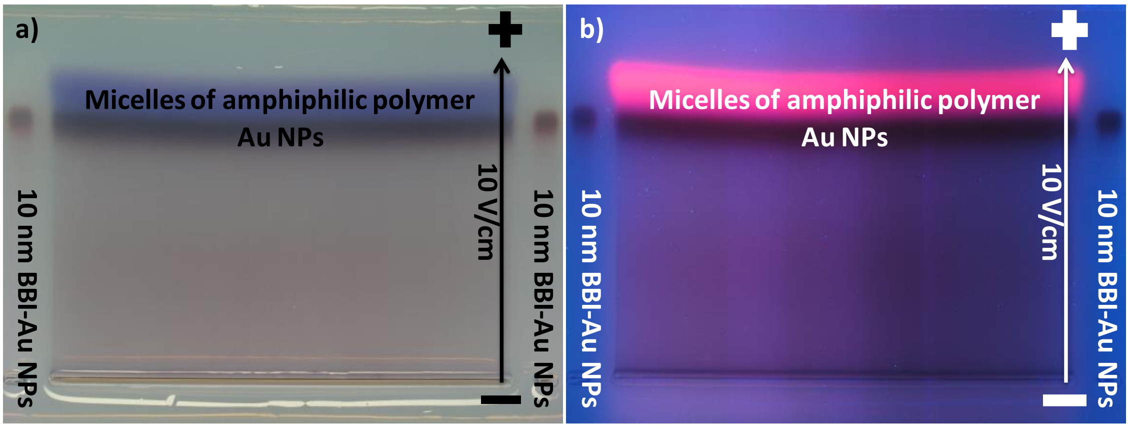

Figure S8: These images were taken from one gel filled with the solution of Au NPs that also contained polymer micelles.[15] a) Image taken under visible light, in which the micelles are identifiable by the purple and the Au NPs by the deep red band. b) Image taken under UV light, in which the fluorescenece of the modified polymere micelles is obvious. The images were taken after 60 min running time within an electric field of 10 V/cm. The lanes on the very left as well as on the very right side correspond to commercial Au NPs (British Biocell International, # EM.GC10) with a size of 10 nm, which serve as internal reference standard (ligand was exchanged with bis(*p*-sulfonatophenyl)phenylphosphine dihydrate dipotassium salt (Strem Chemicals, # 15-0463)).[16] "+" and "-" signs indicate the direction of the electric field. The particles were loaded into the gel at the lower edge.

To extract the separated Au NPs, the corresponding band was cut out of the gel, put into a dialysis membrane (MWCO = 50 kDa, Roth, # 1893.1) and filled up with 0.5× TBE buffer.[17] The sealed hose was placed in the gel electrophoresis device so that it was completely covered with buffer. A voltage of

100 V was applied for ca. 20 min to allow the negatively charged Au NPs to migrate out of the gel. The TBE buffer in the dialysis membrane was then enriched with particles which were concentrated again in a centrifuge filter (3000 rpm, MWCO = 100 kDa, Satorius Stedim, # VS2042). One must note that in some cases small pieces of gel were left in the sample after the gel electrophoresis. In this case the sample was again purified with a 0.22 µm syringe filter.

The particles were further purified *via* size exclusion chromatography (SEC) within an Agilent 1100 HPLC system with fluorescence and absorbance detectors. The sample was run through a self-packed column (Sephacryl S-300 HR, GE Health Care, # 17-0599-01) with sodium borate buffered saline at pH 9 (SBBS9, 50 mM sodium borate and 100 mM NaCl adjusted to pH 9 with NaOH) as mobile phase.[6] Figure S9 shows an illustrative chromatogram in which the absorbance at 220 nm (corresponding to the polymer shell) and 520 nm (corresponding to Au NPs), and the fluorescence intensity at 475 nm (corresponding to AMQB) are shown.

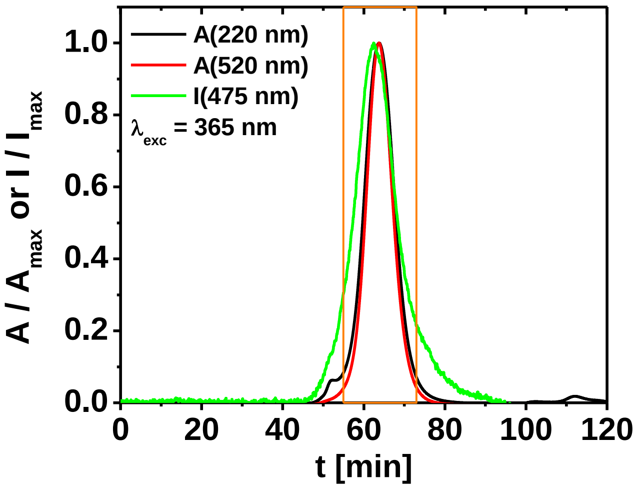

Figure S9: Size exclusion chromatogram of Au NPs coated with negatively charged polymer functionalized with AMQB and CV. All graphs were normalized on 1. The fraction indicated by the orange frame was collected for further processing. That the fluorescence band (green line) is shifted to an earlier appearance to a small extend might be due to the fact that not all particles are carrying the same number of dye molecules.

Afterwards, the sample was concentrated again and run over a desalting column (PD10, GE Healthcare, # 17085101) with Milli-Q water as mobile phase followed by two additional washing steps in centrifuge filters to ensure the separation of ions from the sample volume.

1.5.2 Purification of positively charged Au NPs

In contrast to negatively charged Au NPs the positively charged ones were not purified *via* gel electrophoresis, as the particles did not migrate into the gel, probably due to interactions with the agarose matrix. Also, SEC did not prove as an efficient purification method for the present particles as it was accompanied by significant losses of sample. Thus, the positively charged Au NPs, whether functionalized *via* the crosslinker sulfo-SMCC or SM(PEG)$_2$, were purified from empty polymer micelles

and undesirable salt contents by at least two runs in desalting columns (PD10, GE Healthcare, # 17085101) with Milli-Q water as mobile phase. Hereby, the tailing gave information about the degree of purity. Afterwards, the samples were further purified from smaller molecules and ions by at least 5 washing steps in centrifuge filters (3000 rpm, MWCO = 100 kDa, Satorius Stedim, # VS2042).

1.6 UV/Vis absorption spectroscopy

The concentration of the Au NPs suspensions was determined *via* UV/Vis absorption spectroscopy using Lambert-Beer's law (see Chapter 1.1 and Equation S1). Some absorption spectra are shown in Figure S10.

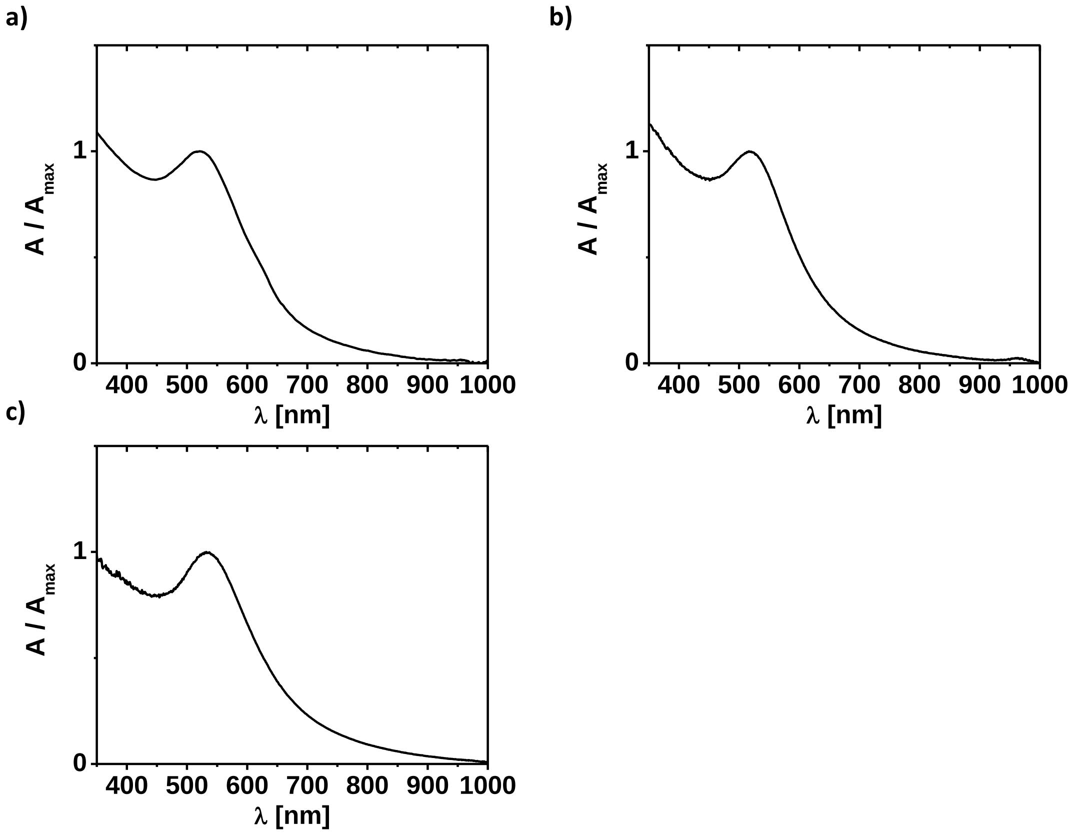

Figure S10: Normalized absorption spectra of a) negatively charged Au NPs functionalized with AMQB and CV, b) positively charged Au NPs functionalized with amino-MQAE *via* SMCC, and c) positively charged Au NPs functionalized with amino-MQAE and CV *via* SM(PEG)$_2$.

2 Examples for ion-sensing
2.1 Measurement set-up
2.2 H^+-sensing with SNARF attached to negatively charged polymer with spacers
2.3 H^+-sensing with OG attached to negatively charged polymer
2.4 Cl^--sensing with MAQE attached to negatively charged polymer with spacers
2.5 Zn^{2+}-sensing with AMQB attached to negatively charged polymer
2.6 Cl^--sensing with MAQE attached to positively charged polymer with spacers
2.7 Summary of sensing concept

2.1 Measurement set-up

All fluorescence measurements were recorded with a Fluorolog-3 (model FL3-22) by Horiba. The slit widths for excitation and emission light were always set to 5 nm each. The integration time was always set to 0.1 s per 1 nm (increment: 1 nm). All samples were measured within a 3-window ultra-micro-cuvette (Hellma Analytics, # 105-252-15-40) with at least 30 µL sample volume. Au NPs solution and analyte solution were mixed (1:1) in the cuvette prior to each measurement. All concentrations are depicted as true concentration in the cuvette, not as concentration of the respective stock solution. Stock solutions were always prepared prior to a measurement series as dilution from a higher concentrated stock solution with fresh Milli-Q water. In case of Zn^{2+} reponsive Au NPs, $Zn(NO_3)_2{\cdot}6H_2O$ (Sigma-Aldrich, # 228737) and in case of Cl^- reponsive Au NPs, NaCl (Roth, # HN00.2) were chosen to prepare the analyte solutions.

2.2 H^+-sensing with SNARF attached to negatively charged polymer with spacers

These data are taken from a previous publication and we refer to this publication for the experimental procedures.[10] The response curves are shown in Figure S11. The presented effect, that ions in solution are attracted or repulsed by colloidal NPs depending on the sign of charge does not compellingly have to be a disadvantage. It offers the possibility to tune the sensitivity of fluorescent dyes linked to NPs regarding the local concentration of ions. This is shown in the present example for the pH-sensitive dye seminaphtharhodafluor (SNARF).[10] Once linked to the surface of negatively charged Au NPs with spacer molecules of various lengths, the dye responds to its local pH, *i.e.* to the local concentration of hydronium ions. As the analytes are attracted by the NP surface, a low pH is connoted by SNARF molecules linked with a short spacer. Employment of a long spacer results in a readout signal similar to that of the free dye. With this in mind, the reponsive region of the sensor device can be tuned to be in a certain range of pH.

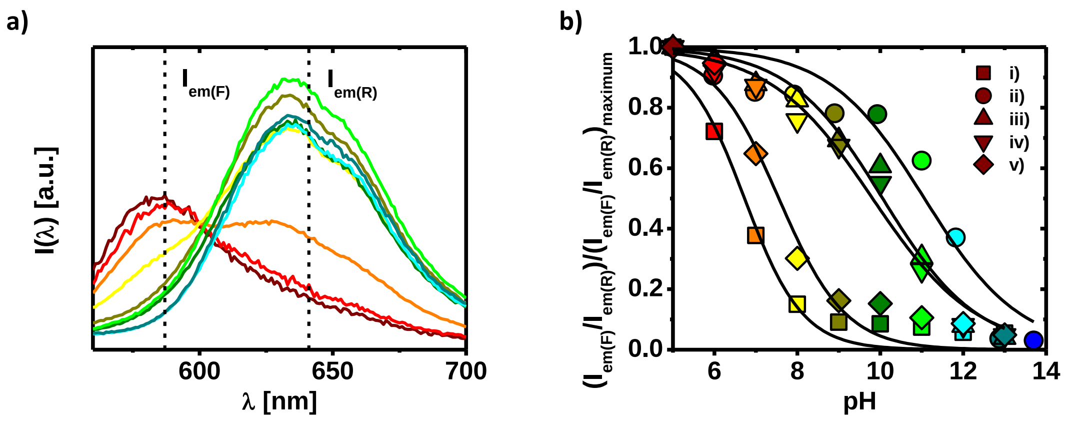

Figure S11: a) Fluorescence emission spectrum $I(\lambda)$ of the free H^+-reponsive fluorophore seminaphtharhodafluor (SNARF) conjugated to 2 kDa poly(ethylene glycol) (PEG) in dependence of the bulk H^+ concentration $c(H^+)$ [M] (given as pH = -log(c(H$^+$)/M)), upon excitation at λ_{ex} = 540 nm. SNARF is a ratiometric fluorophore (F) which (in a simplified way of description) integrates the reference fluorophore (R) in the ion-reponsive fluorophore (F). Two emission peaks can be distinguished: $I_{em(F)}$ at $\lambda_{em(F)}$ = 587 nm and $I_{em(R)}$ at $\lambda_{em(R)}$ = 641 nm. b) The response to bulk pH is given as ratio $I_{em(F)}$ /$I_{em(R)}$. The normalized response I_{norm} = ($I_{em(F)}$/$I_{em(R)}$)/($I_{em(F)}$/$I_{em(R)}$)$_{maximum}$ is shown for the following geometries: SNARF not bound to NPs (i), and SNARF linked to the NP surface with PEG spacers (S) to the surface of negatively charged NPs with spacers of molecular weight 0.1 kDa (ii), 2 kDa (iii), 6 kDa (iv), 10 kDa (v). This graph is adopted with permission from Zhang *et al.*[10], Copyright (2010) John Wiley and Sons. This graph refers to Figure 1A in the main manuscript.

2.3 H^+-sensing with OG attached to negatively charged polymer

Oregon green (OG) is another pH-responsive fluorophore. Data are shown in Figure S12 and Figure S13. These data have been taken from a previous publication and we refer to this publication for further experimental details.[11]

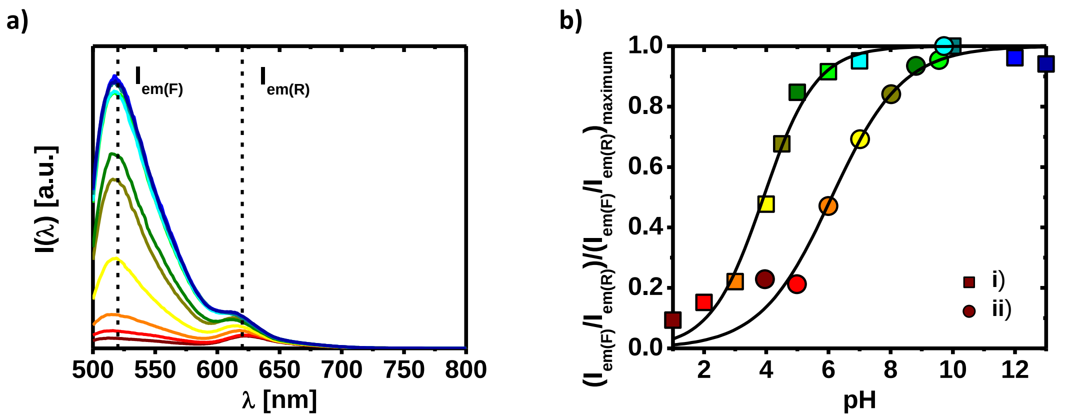

Figure S12: a) Fluorescence emission spectrum $I(\lambda)$ of the H^+-reponsive fluorophore Oregon Green (F) mixed together with the reference fluorophore (R) ATTO-590 in dependence of the bulk H^+ concentration $c(H^+)$ [M] (given as pH = -log(c(H$^+$)/M)), upon excitation at λ_{ex} = 488 nm. Two emission peaks originating from Oregon Green and ATTO-590 can be distinguished with $I_{em(F)}$ at $\lambda_{em(F)}$ = 520 nm and $I_{em(R)}$ at $\lambda_{em(R)}$ = 620 nm, respectively. b) The response to bulk pH is given as ratio $I_{em(F)}$ /$I_{em(R)}$. The

normalized response I_{norm} = $(I_{em(F)}/I_{em(R)})/(I_{em(F)}/I_{em(R)})_{maximum}$ is shown for the following geometries: Mixture of Oregon Green and ATTO-590 not bound to NPs (i), and Oregon Green and ATTO-590 linked without spacers (S) directly to the surface of negatively charged NPs (ii). This graph is adopted with permission from Zhang *et al.*[11], Copyright (2011) John Wiley and Sons. This graph refers to Figure 1B in the main manuscript.

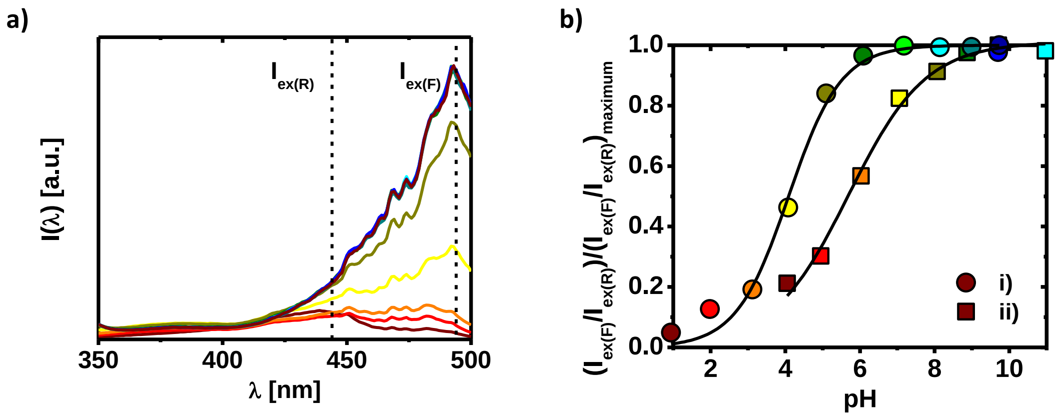

Figure S13: a) Fluorescence excitation spectrum $I(\lambda)$ of the H^+-reponsive fluorophore Oregon Green (F) in dependence of the bulk H^+ concentration $c(H^+)$ [M] (as given as pH = $-\log(c(H^+)/M)$), upon recording the emission at λ_{em} = 518 nm. This demonstrates that sensing can be performed not only *via* emission but also *via* excitation spectra. Oregon green is a ratiometric fluorophore which (in a simplified way of description) integrates the reference fluorophore (R) in the ion-reponsive fluorophore (F). Two excitation peaks can be distinguished with $I_{ex(F)}$ at $\lambda_{ex(F)}$ = 494 nm and $I_{ex(R)}$ at $\lambda_{ex(R)}$ = 444 nm, respectively. b) The response to bulk pH is given as ratio $I_{ex(F)}$ /$I_{ex(R)}$. The normalized response I_{norm} = $(I_{ex(F)}/I_{ex(R)})/(I_{ex(F)}/I_{ex(R)})_{maximum}$ is shown for the following geometries: Oregon Green not bound to NPs (i), and Oregon Green linked without spacer (S) directly to the surface of negatively charged NPs (ii). This graph is adopted with permission from Zhang *et al.*[11], Copyright (2011) John Wiley and Sons.

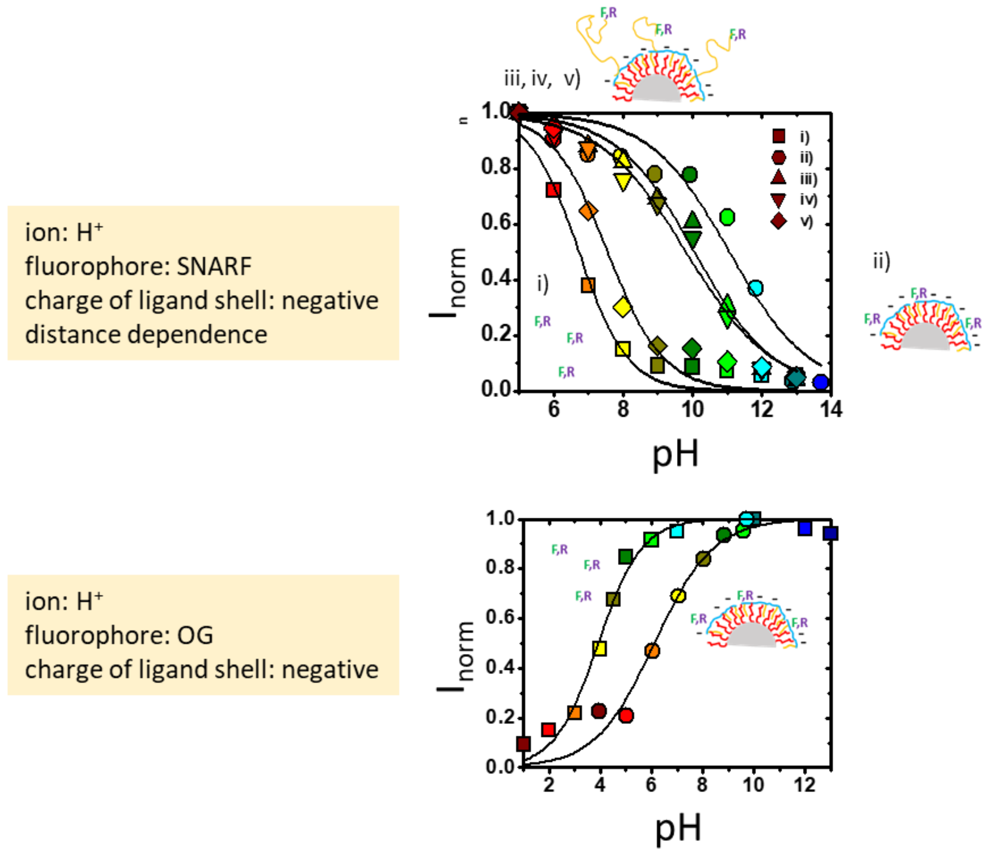

Figure S14: Compilation of all Figures concerning H$^+$ sensing. This is a part of Figure 1 from the main paper with higher magnification and comprises data from Figure S11 and Figure S12.

2.4 Cl$^-$-sensing with MAQE attached to negatively charged polymer with spacers

The fluorescence behavior of bare amino-MQAE in response to various Cl$^-$ concentrations was evaluated prior to this study.[8] A fluorescence quenching (exponential decay) down to a saturation plateau of approximately 10% for 140 mM NaCl was observed as compared to the fluorescence intensity at 0 mM NaCl (≙ 100%). For negatively charged Au NPs functionalized with amino-MQAE directly at the NP surface the fluorescence was only quenched down to approximately 60% for 140 mM NaCl compared to 0 mM NaCl (≙ 100%), indicating a lower local (at the position of the dye) Cl$^-$ concentration compared to the bulk concentration due to the repulsion of Cl$^-$ ions by the negatively charged NP surface. Thus, in this previous study it had been shown, that the closer the MQAE is located to the surface of negatively charged NPs, the less reponsive it is to Cl$^-$.[8] This was explained by repulsion of Cl$^-$ by the negatively charged NP surface. Distance between MQAE and the NP surface was varied by using PEG spacers. The experimental data from Riedinger *et al.* are reproduced in Figure S15.

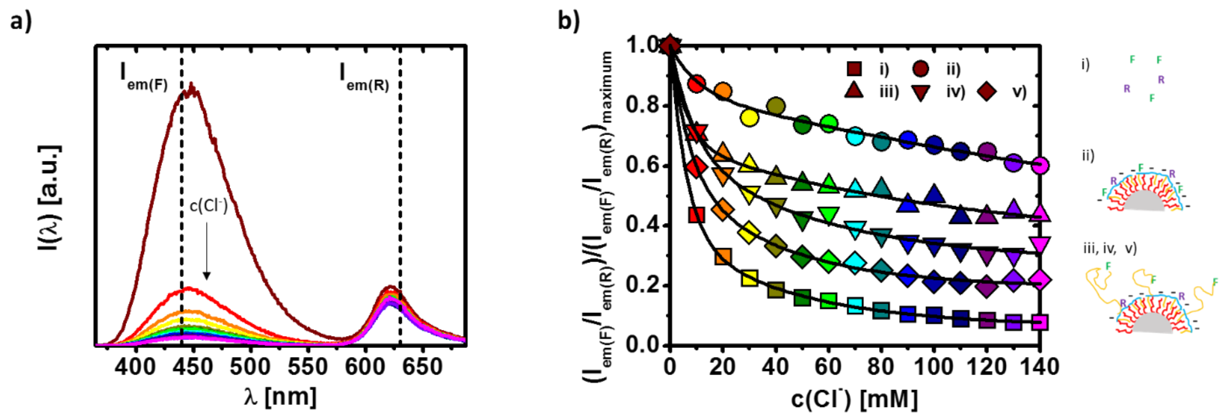

Figure S15: a) Fluorescence emission spectrum $I(\lambda)$ amino-MQAE (F) mixed together with the reference fluorophore CVP (R) in dependence of the bulk Cl^- concentration $c(Cl^-)$, upon excitation at λ_{ex} = 350 nm. Two emission peaks originating from amino-MQAE and CVP can be distinguished with $I_{em(F)}$ at $\lambda_{em(F)}$ = 440 nm and $I_{em(R)}$ at $\lambda_{em(R)}$ = 630 nm, respectively. b) The response to bulk Cl^- is given as ratio $I_{em(F)}$ /$I_{em(R)}$. The normalized response $I_{norm} = (I_{em(F)}/I_{em(R)})/(I_{em(F)}/I_{em(R)})_{maximum}$ is shown for the following geometries: (i) Mixture of free amino-MQAE and CVP (*i.e.* not bound to NPs; these data correspond to the spectra shown in a)). (ii) MQAE and CV directly integrated in the polymer surface of the negatively charged NPs. (iii - v) MQAE linked with PEG spacers with molecular weight (iii) 3 kDa, (iv) 5 kDa, and (v) 10 kDa to the surface of negatively charged NPs. CV was directly integrated into the polymer coating. This graph is adopted with permission from Riedinger *et al.*[8], Copyright (2010) John Wiley and Sons. This graph refers to Figure 1C in the main manuscript.

2.5 Zn^{2+}-sensing with AMQB attached to negatively charged polymer

The response of the synthesized NPs, with AMQB integrated into the negatively charged polymer surface, to increasing Zn^{2+} ion concentrations was studied through the Zn^{2+}-dependent changes of their fluorescence emission. To this end, the NPs (after all purification steps) were immersed in aqueous solution under addition of different amounts of Zn^{2+}, and the respective fluorescence spectra were recorded. The concentration of Au NPs in the cuvette was c_{NP} = 0.175 µM. As a control, the response of the free dyes was measured. Presence of Zn^{2+} leads to an increase of fluorescence of AMQB. For NPs modified with negatively charger polymer with integrated AMQB and CV (Figure S16d) the fluorescence of AMQB increases less with increasing Zn^{2+} concentration as compared to the fluorescence of free AMQB and CVP (Figure S16b). Also, the location of the emission peak was shifted by about 15 nm towards smaller wavelengths (Figure S16c). Moreover, a plateau was reached for much smaller Zn^{2+} concentrations in case of the NP-dye conjugate. This goes hand in hand with the assumption that the (double-positively charged) Zn^{2+} ions are attracted by the negatively charged NP surface, thus gaining a much higher local concentration at the NP surface as compared to the bulk. Hence a state of saturation is reached for smaller bulk Zn^{2+} concentrations.

It should be mentioned that the reponsive region matches partly with the concentration of Au NPs, thus Zn^{2+} ions and Au NPs are present within equimolar amounts in this region. Another point to be mentioned is that actually two AMQB molecules are required to capture one Zn^{2+} ion (2:1 stoichiometry) for efficient fluorescence amplification.[9] Thus, two scenarios theoretically might occur. The first is that two AMQB molecules are located at the polymer backbone of the Au NP in very close

proximity and the flexibility of the polymer allows for an efficient capture of Zn^{2+} ions. The second is that two Au NPs get into close proximity and a Zn^{2+} ion is captured by AMQB molecules of these two individual particles. The latter scenario seems to be unlikely due to the electrostatic repulsion between Au NPs, though the present Zn^{2+} content might attenuate the repulsion due to screening of the surface charges.

This example proves that when the Zn^{2+}-reponsive dye is placed close to surface of negatively charged NPs, the sensitivity of the system increases significantly due to the pre-concentration effect of Zn^{2+}. It demonstrates once more, that by placing ion-reponsive fluorophores in the vicinity of charged NP surfaces, the reponsive range of the fluorophores can be modified, due to the different local ion concentrations as compared to bulk. Such pre-concentration can be used to increase sensitivity, as presented here for the case of Zn^{2+}.

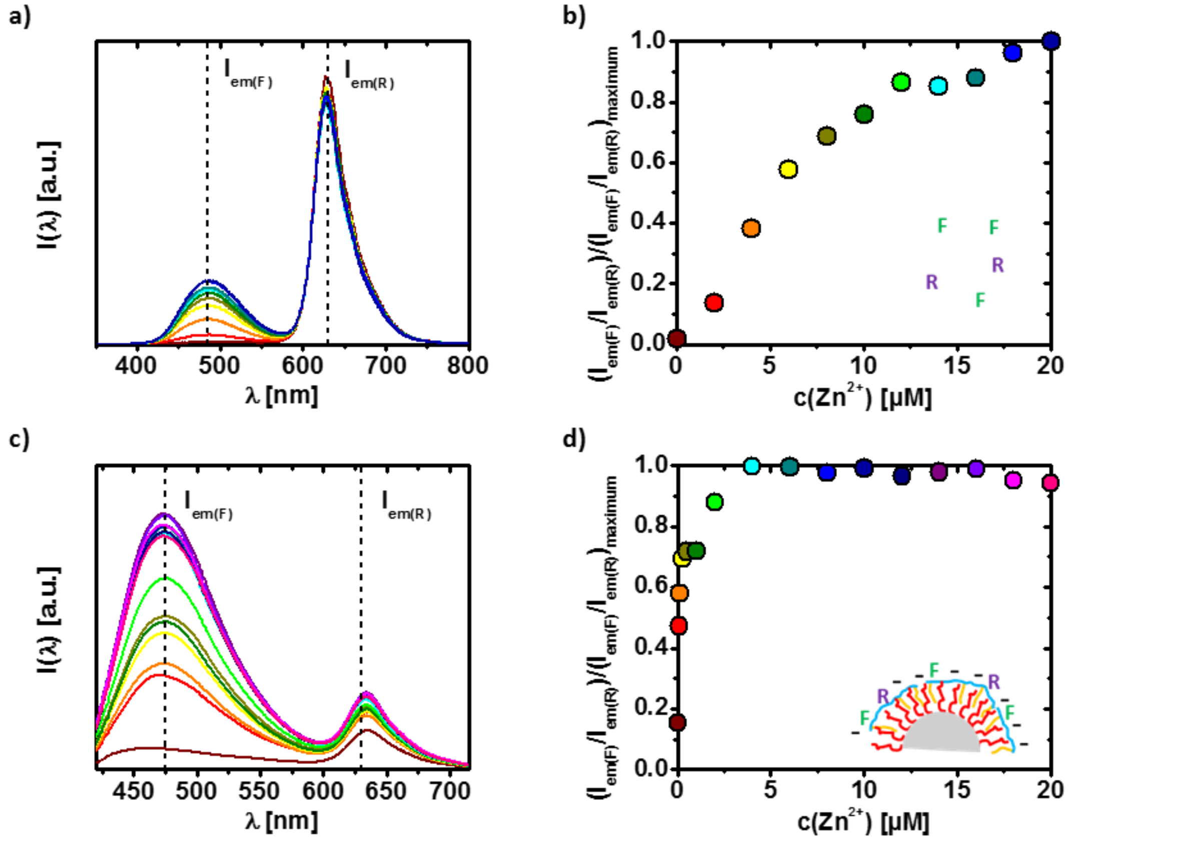

Figure S16: a) Fluorescence emission spectrum $I(\lambda)$ of the Zn^{2+}-reponsive fluorophore AMQB (F) mixed together with the reference fluorophore (R) CV in water/ethanol (1:1) in dependence of the bulk Zn^{2+} concentration $c(Zn^{2+})$, upon excitation at λ_{ex} = 340 nm with c(AMQB) = 0.2 mM. Two emission peaks originating from AMQB and CV can be distinguished with $I_{em(F)}$ at $\lambda_{em(F)}$ = 485 nm and $I_{em(R)}$ at $\lambda_{em(R)}$ = 630 nm, respectively. b) The response of a mixture of free amino-AMQB and CVP (i.e. not bound to NPs) to bulk Zn^{2+} of is given as ratio $I_{em(F)}$ /$I_{em(R)}$. The normalized response I_{norm} = $(I_{em(F)}/I_{em(R)})/(I_{em(F)}/I_{em(R)})_{maximum}$ as derived from the spectra displayed in a) is shown. The color of the data points corresponds to the color of the spectra. An amplification of the intensity by a factor of ≥ 50 times is observed. The reponsive region extends over the whole set of evaluated Zn^{2+} concentrations. c) Fluorescence intensity $I(\lambda)$ of Au NPs functionalized with negatively charged polymer with integrated AMQB and CV suspended in water for various Zn^{2+} concentrations. λ_{exc} = 365 nm, c_{NP} =

0.175 µM. The emission peak of AMQB appears at $\lambda_{em(F)}$ = 475 nm (15 nm blue shifted as compared to the free dye) and the emission peak of CP appears at $\lambda_{em(R)}$ = 630 nm. Although the emission of CP is partly overlapping with the emission of AMQB it serves as a suitable control for normalization. d) The response to bulk Zn^{2+} is given as ratio $I_{em(F)}/I_{em(R)}$. The maximum intensities $I_{em(F)}$ at approximately 475 nm (averaged from 470 nm to 480 nm) and the corresponding CV intensities $I_{em(R)}$ at 630 nm (averaged from 625 nm to 635 nm) are taken from c). The normalized response I_{norm} = $(I_{em(F)}/I_{em(R)})/(I_{em(F)}/I_{em(R)})_{maximum}$ is shown. An increase of the intensity upon the addition of Zn^{2+} up to approximately 6.5 times is observed. The reponsive region extends over 0 µM to 5 µM. This graph refers to Figure 1D in the main manuscript.

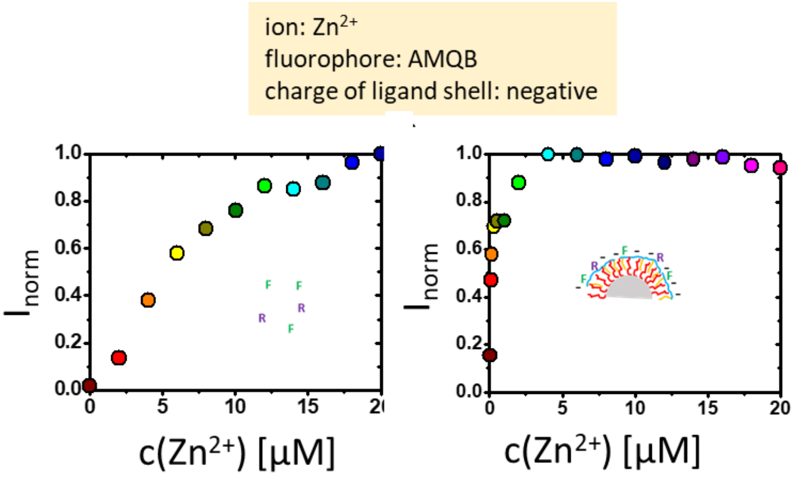

Figure S17: Compilation of all Figures concerning Zn^{2+} sensing. This is a part of Figure 1 from the main paper with higher magnification and comprises data from Figure S16.

2.6 Cl^--sensing with MAQE attached to positively charged polymer with spacers

To verify that MQAE is applicable also with positively charged NPs, the surfaces of the Au NPs were coated with a positively charged polymer PTMAEMA-*stat*-PLMA. Amino-MQAE was attached to the sulfhydril groups of the polymer surface using the crosslinker sulfo-SMCC. The length of one SMCC molecule was assumed to be approximately 0.83 nm, which (as the linker is not rigid) results in an average distance of the fluorophore to the NP surface of 1/2·0.83 nm ≈ 0.4 nm. Comparison of the response curves for free amino-MQAE/CVP (Figure S18a,b) to the ones for MQAE and CV integrated to the positively charged surface of NPs (Figure S18c,d) shows that proximity of MQAE to the positively charged NP surface did not enhance its sensitivity to detect Cl^-. On the one hand the decay is nearly linear and on the other hand a saturation plateau is not reached within the considered range of Cl^- concentrations. A possible reason for this behavior is that the spacer arm length of SMCC (8.3 Å) is long enough to bridge the accumulated Cl^- ions at the Au NP surface. In other words: The dimension of the Cl^- cloud is smaller compared to the distance between NP surface and dye location. Thus, compared to negatively charged NPs, more Cl^- is needed in bulk to reach a saturation plateau. The simplistic assumption of pre-concentration of Cl^- ions close to the NP surface alone can't explain the effect.

To further investigate the effect of the underlying positively charged NP surface, MAQE was linked not directly, but via a spacer molecule of approximately the double length (1.76), namely succinimidyl-([*N*-

maleimidopropionamido]-2 ethylene glycol ester (SM(PEG)$_2$) to the surface of the positively charged NPs. The comparison of both responses (Figure S18c,d and Figure S18e,f) shows clearly that the first hybrid (Figure S18c,d) using a short spacer molecule (SMCC)) is much more reponsive to low Cl^- concentrations, as a higher fluorescence decrease is observed for the same Cl^- bulk concentration. In contrast, in the second hybrid (Figure S18e,f), which has the dye further away from the NP surface, significantly lower local Cl^- concentration must be present around the responsive dye. Here, the fluorescence intensity was quenched down to a plateau of about 70% compared to the intensity without NaCl (≙ 100%). Thus, compared to the shorter spacer SMCC (8.3 Å) the decrease within the range of the longer spacer SM(PEG)$_2$ (17.6 Å) is even less distinct. This underlines the assumption that Cl^- ions accumulated at the NP surface infiltrate the location of the dye molecules. In summary, Figure S18 clearly demonstrates that upon conjugation to the surface of positively charged NPs, the working range of the Cl^--reponsive fluorophore is extended. While the free fluorophore is most reponsive (biggest slope in the response curve) to changes in the Cl^- concentrations between ca. 0 - 40 mM, the most reponsive range of operation for the Cl^--reponsive fluorophore attached to the NPs is for Cl^- concentrations between 40 and 150 mM. Thus, while the effect of attaching the Cl^--reponsive fluorophore to the surface of positively charged NPs could not be quantitatively predicted by "intuition", it still clearly allows for shifting the working point, *i.e.* the concentration range which can be detected.

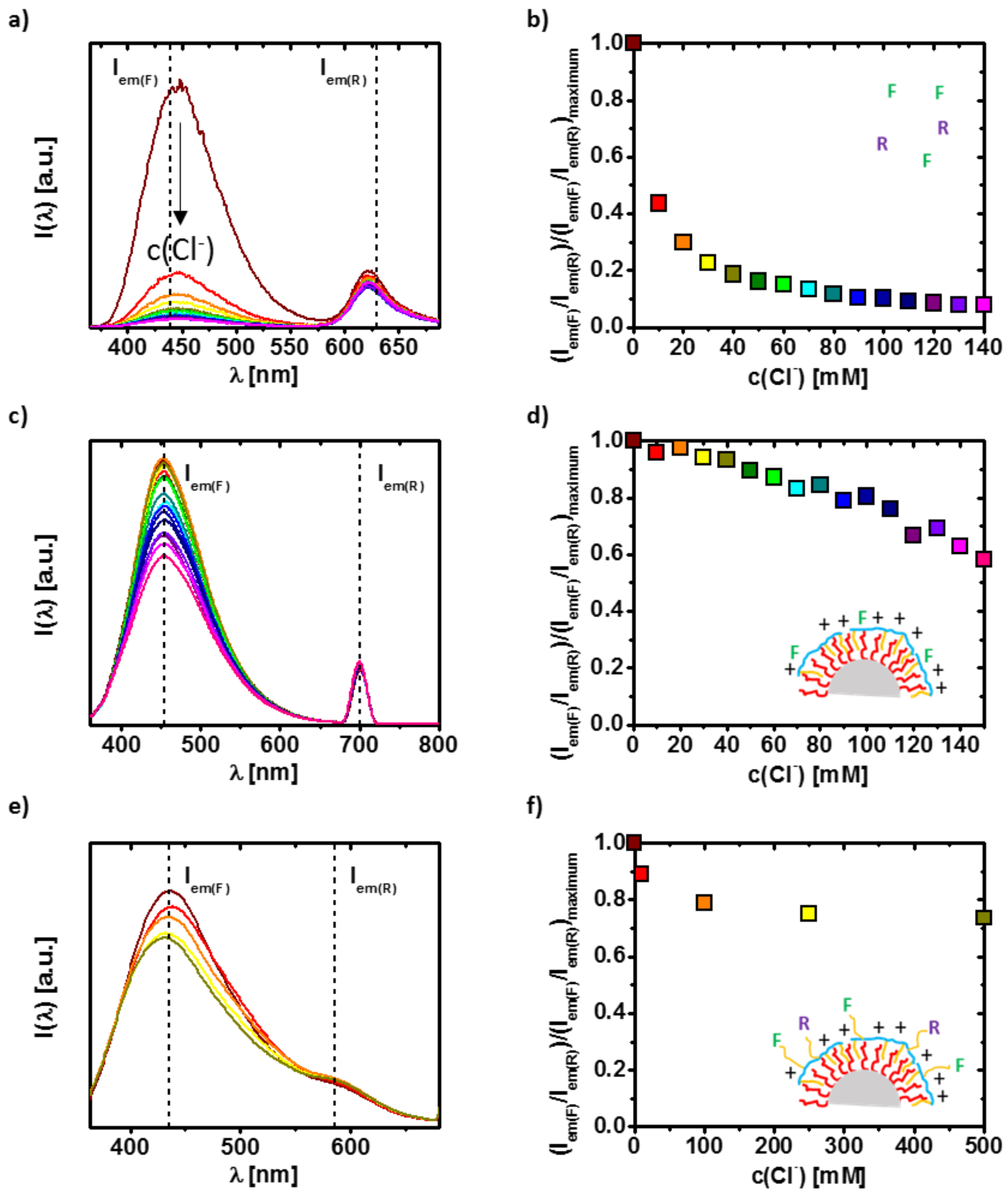

Figure S18: a) Fluorescence emission spectrum $I(\lambda)$ of the Cl^--reponsive fluorophore amino-MQAE (F) mixed together with the reference fluorophore CV (R) in dependence of the bulk Cl^- concentration $c(Cl^-)$, upon excitation at λ_{ex} = 350 nm. Two emission peaks originating from amino-MQAE and CV can be distinguished with $I_{em(F)}$ at $\lambda_{em(F)}$ = 440 nm and $I_{em(R)}$ at $\lambda_{em(R)}$ = 630 nm, respectively. b) The response to bulk Cl^- is given as ratio $I_{em(F)}$ /$I_{em(R)}$. The normalized response $I_{norm} = (I_{em(F)}/I_{em(R)})/(I_{em(F)}/I_{em(R)})_{maximum}$ is shown for a mixture of free amin-MQAE and CV, which are not bound to NPs. The data points are derived from the spectra shown in a), and their colors correspond to the colors of the spectra. c) Fluorescence intensity $I(\lambda)$ of positively charged Au NPs functionalized with amino-MQAE via SMCC suspended in water for various Cl^- concentrations. The arrow indicates the direction of increasing Cl^- concentration. λ_{exc} = 350 nm, c_{NP} = 0.1 µM. The emission peak of amino-MQAE appears at $\lambda_{em(F)}$ = 455 nm (15 nm red shifted compared to the free dye). In this sample no reference dye was used for normalization. Instead, the $2 \cdot \lambda_{exc}$ scattering peak observed in the fluorometer at twice the excitation wavelength at $\lambda_{sc(R)}$ = "$\lambda_{em(R)}$" = 700 nm served for normalization. d) The response to bulk Cl^- is given as ratio $I_{em(F)}$ /$I_{em(R)}$. The intensities extracted from the spectra shown in c) were divided by the corresponding $2 \cdot \lambda_{exc}$ peak intensity at 700 nm then the data points were normalized in a way that the

value 1 corresponds to 0 mM Cl^-. The resulting normalized response I_{norm} = $(I_{em(F)}/I_{em(R)})/(I_{em(F)}/I_{em(R)})_{maximum}$ is shown. An intensity quenching by a factor of approximately 1.7 is observed. The sensitive region extends over the whole set of evaluated Cl^- concentrations. e) Fluorescence intensity $I(\lambda)$ of positively charged Au NPs functionalized with amino-MQAE and CV *via* SM(PEG)$_2$ suspended in water for various Cl^- concentrations. λ_{exc} = 350 nm, c_{NP} = 0.1 µM. The emission peak of amino-MQAE appears at $\lambda_{em(F)}$ = 435 nm (5 nm blue shifted compared to the free dye) and the emission peak of CV appears as shoulder at $\lambda_{em(R)}$ = 585 nm. Although the emission of CV is partly overlapping with the emission of amino-MQAE, it serves as a suitable control for normalization. f) The response to bulk Cl^- is given as ratio $I_{em(F)}$ $/I_{em(R)}$. The normalized response I_{norm} = $(I_{em(F)}/I_{em(R)})/(I_{em(F)}/I_{em(R)})_{maximum}$ as derived from the spectra as depicted in e) is shown. An intensity quenching by a factor of approximately 1.3 is observed. While one would have expected a steeper response of the Cl^--reponsive fluorophore upon attachment to the positively charged NPs the experimental data demonstrate that this is not the case. These graphs refer to Figure 1E in the main manuscript.

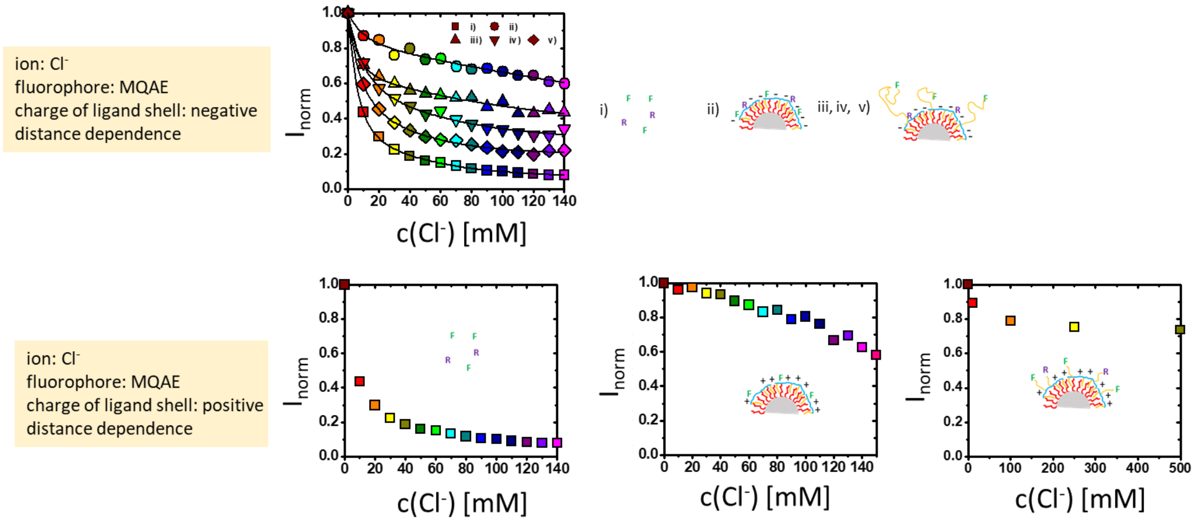

Figure S19: Compilation of all Figures concerning Cl^- sensing. This is a part of Figure 1 from the main paper with higher magnification and comprises data from Figure S15 and Figure S18.

2.7 Summary of sensing concept

As state of the art it was described how the presence of a NP surface close to analyte-reponsive fluorophores can change their response to the analyte.[8,10] So far, this fact has been considered as a drawback or a potential source of error in the field of sensors based on NPs, which had to be corrected properly in the calibration curve. The idea now is to exploit this effect to improve the analytical performance of analyte-reponsive fluorophores, *i.e.* to control the working range / the sensitivity of analyte-reponsive fluorophores by linking them to the surface of NPs controlling the following parameters: The polarity of the NP surface (sign of charge, charge density, hydrophilic/hydrophobic behavior), and the distance of the fluorophore (F) to the NP surface as mediated by molecular spacer molecules (S). In this way, the charged NP would act not as a simple passive carrier, but as an active element in the sensing system. In the case of ion-reponsive fluorophores, sensitivity can be tuned by the sign of the surface charge of the NPs. In case the NP is oppositely charged, in general the local

concentration of the ion to be detected will be increased at the NP surface and sensitivity in detection is increased, *i. e.* the NP surface allows for (nonspecific) preconcentration of oppositely charged ions. In the opposite direction, in case the working range should be extended, linkage of the ion-reponsive fluorophore to a NP surface with opposite charge will lead to local (nonspecific) depletion of the ions to be detected and thus to intentionally reduced sensitivity. The effect can be further modulated by the charge density of the NP surface (the bigger the charge density, the higher the effect) and the distance of the fluorophore to the NP surface (the closer the fluorophore, the higher the effect). It is important to point out, that it is not possible up to now to quantitatively predict the tuning or the working point of ion-reponsive fluorophores by attaching them to the surface of NPs. Accordingly, the details rely on experimental fine-tuning. Besides local preconcentration / depletion of ions in the context of ion-reponsive fluorophores, the same effect can also be used for local preconcentration / depletion of polar molecules, by changing the hydrophilicity/hydrophobicity of the NP surface. The less polar the NP surface, the higher the preconcentration effect of apolar analytes will be (note that solvents need to be chosen appropriately in order to maintain colloidal stability of the NPs). In this way, attaching analyte-reponsive fluorophores (F) to the surface of NPs, by varying the charge (C) of the NP surface and by varying the distance between the fluorophore and the NP surface *via* molecular spacers (S), the working point (*i.e.* the concentration range of the analyte at which the fluorophore responds with the highest changes in optical output signal) can be experimentally tuned.

3 Ion-response simulations for negatively charged surfaces

While the data shown in Figure S15 concerning Cl^- detection close to the surface of charged NPs can be understood in principle by Debye Hückel screening, though the effect is less obvious for the data shown in Figure S18. For a more elaborate tuning of the working point of ion-reponsive fluorophores close to NP surfaces a more quantitative analysis is required. The general problem hereby is, that the geometry of the NPs is not well defined. Even if assuming the NP core to be a perfect sphere, the polymer coating does not lead to a homogeneous surface. The thickness of the polymer shell is in fact rather a distribution than a precise value, and consequently also the distance of the fluorophores with respect to the NP surface is distributed around a mean value. In order to describe the geometry of the NPs, the parameters shown in Figure S20 were used. An Au NP core of radius r_c is capped by a layer of dodecanethiol and then overcoated by PMA.[5] Note that here the discussion is done in terms of radius r, whereas in the synthesis part discussion was done in terms of diameter $d = 2 \cdot r$. The organic coating forms a hydrophobic layer $r_c < r < r_{eff}$, formed by the dodecanethiol and the hydrophobic alkylamine side-chains of the amphiphilic polymer. This is followed by a hydrophilic layer formed by the polymer backbone of PMA, which is bearing the negative charge of the carboxyl groups, $r_{eff} < r < r_p$. PEG chains of various molecular weight are used as molecular spacer, and the ion-sensitive fluorophore (F = MQAE) is covalently bound to the PEG terminus. The distance of the fluorophore to the NP center is denoted as r_F.

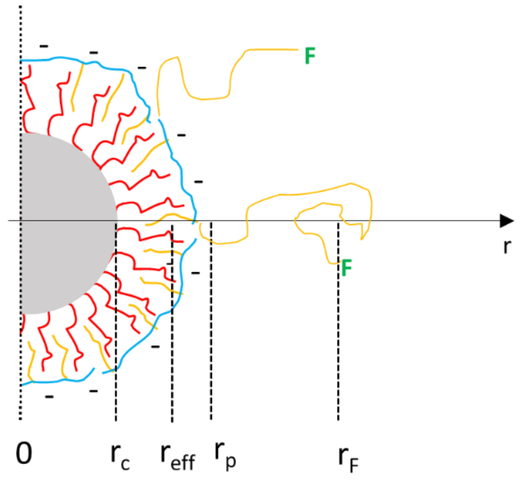

Figure S20: Sketch of the NP geometry assumed for the theoretical calculations. Here, r_c is the radius of the Au core, and r_{eff} and r_p denote the inner and outer bound a hydrocarbon chain layer (red) and the resulting amphiphilic polymer coating with negative chrged (orange, blue), respectively, with a layer thickness of $r_p - r_{eff} = 1$ nm and a homogeneous charge with number density ρ_{layer}. The hydrocarbon ligand layer itself is assumed to have a thickness of $r_{eff} - r_c = 1$nm. The anchor point of the PEG backbone is assumed to be near r_p (see text). The position of the fluorophore attached to the PEG spacer is denoted as and r_F. The reference fluorophore in case of absence of a spacer is assumed to be located near the charged layer.

The ion-responsive fluorophore has a fluorescence emission $I(c)$ as a function of the local ion concentration $c(r)$, which is normalized to the fluorescence emission without the presence of ions (c = 0). The fluorescence readout $\langle I \rangle$ will thus depend on the concentration distribution of the ions to which the ion-responsive fluorophore responds, but it will also depend on the distance distribution of the fluorophore to the NP surface. The location of the fluorophores, which is determined by the PEG

spacer, is expressed in terms of the probability distribution $P_e(r)$ of finding the PEG end at a distance r from the NP center. The total fluorescence thus is estimated as

$$\langle I \rangle = \frac{\int_{r_C}^{\infty} P_e(r_F) \cdot I(c(r_F)) dr_F}{\int_{r_C}^{\infty} P_e(r_F) dr_F} \qquad \text{(Equation S3)}$$

In order to estimate <*I*> for a given geometry and PEG length, numerical access to the distributions $P_e(r)$ and $c(r)$ can be gained via the Poisson Boltzmann equation (PBE)[18] and the Edwards equation, respectively.[19] In Gaussian units, the PBE for the electrical potential φ reads

$$\nabla^2 \varphi = -\frac{4\pi q}{\epsilon}\{\rho_{ext} + \rho_\infty [exp(-Zq\beta\varphi) - exp(+Zq\beta\varphi)]\} \qquad \text{(Equation S4)}$$

where q is the elementary charge and ε the specific dielectric constant. ρ_{ext} (r) is an externally fixed charge number density (*i.e.* the local number of charges per volume) and ρ_∞ is the bulk charge density of the ions to be detected, which is equivalent to the density of counter ions, assuming that a salt was used in which ions and counter ions have the same valence Z (*i.e.* NaCl, $c(Na^+) = c(Cl^-)$, Z = 1). The resulting counter-ion distribution around the NP is determined by the r-dependent potential φ as the solution of the PBE. The given density field $\rho_{ext}(r)$ depends on the radial position r and can be composed by the NP-bound charge distributions, *i.e.* the layer's charge density ρ_{layer} at r_p (Fig. S20), as well as by the polyelectrolyte's (PEG) contribution depending on pH. In Eq. (S4), $\beta = 1/(k_B \cdot T)$, the inverse temperature given by the Boltzmann constant k_B, and the absolute temperature T. The charge density, valence, and bulk ion concentration c_∞ of one component are related by $\rho_\infty = Z \cdot c_\infty$. When introducing the dimensionless potential $u = q \cdot \beta \cdot \varphi$ and the Bjerrum length $l_B = \beta \cdot q^2/\varepsilon$, one may reformulate the PBE for radially symmetric situations in terms of the dimensionless distance $\tilde{r} = r/l_B$ (with r carrying absolute length units) from the NP center as

$$[\frac{2}{\tilde{r}} \partial_{\tilde{r}} + \partial_{\tilde{r}}^2] u(\tilde{r}) = -4\pi[\tilde{\rho}_{ext}(\tilde{r}) - 2\tilde{\rho}_\infty \cdot sinh(Z \cdot u(\tilde{r}))] \qquad \text{(Equation S5)}$$

Here, we give the charge number densities $\tilde{\rho} = l_B^3 \cdot \rho$ per Bjerrum length cube. For the solution of Equation S5 the boundary conditions $\partial_{\tilde{r}} u|_{\tilde{r}=0} = 0$ and $u|_{\tilde{r}\to\infty} = 0$ are required. We use the dimensionless PBE (Equation S5) in order to calculate the field $u(\tilde{r})$ and the corresponding co-ion concentration $c(\tilde{r}) = c_\infty \cdot exp(-u)$. In order to describe a realistic scenario, some parameters have to be obtained from experimental data. In the following, parameters corresponding to the measurements of Cl^- in the vicinity of negatively charged NPs (Figure S15) are used. Let us consider a layer charge density $\rho_{layer} = 1.2/l_B^3$ (i.e. $\tilde{\rho}_{layer}$ = 1.2), which correspond to approximately c_{layer} = 5.8 mol/L. Note that a concentration c = 1 mol/L corresponds to a dimensionless number density of $\tilde{\rho}$ = 0.207. The used value $\tilde{\rho}_{\text{layer}}$ = 1.2 has been derived by matching the theoretical result for <*I*> and the experimental data for c_∞ = 0.14 mol/L for an ion-reponsive fluorophore in case it is located at a distance $r_F = (r_{eff} + r_p)/2$. The experimental data shown in Figure S15 were obtained with Au NPs of a core radius of r_C = 2 nm.[8] The thicknesses of the hydrophobic and hydrophilic layers around the NP cores cannot be precisely determined. The thickness of the organic layer will be bigger than the length of dodecanethiole molecules. The radius r_p of the NPs involving the core, the hydrophobic, and the hydrophilic shell will be less than the hydrodynamic radius ($r_p < r_h$), which had been determined to be r_h = 4.8 nm.[8] In the following, the thickness of the hydrophobic layer (*i.e.* the docecanethiol capping +

hydrophobic side-chains of the amphiphilic polymer) is assumed to be r_{eff} - r_c = 1.0 nm, and the thickness of the hydrophobic layer (*i.e.* the charged polymer backbone) is assumed to be r_p - r_{eff} = 1 nm. Solutions of the PBE using these parameters are shown in Figure S21 by means of the dimensionless potential $u(r)$ for two given bulk concentrations c_∞. Data clearly demonstrate distance dependence of the electrostatic potential, as expected by Debye-Hückel screening due to accumulation of counter ions (here Na^+).

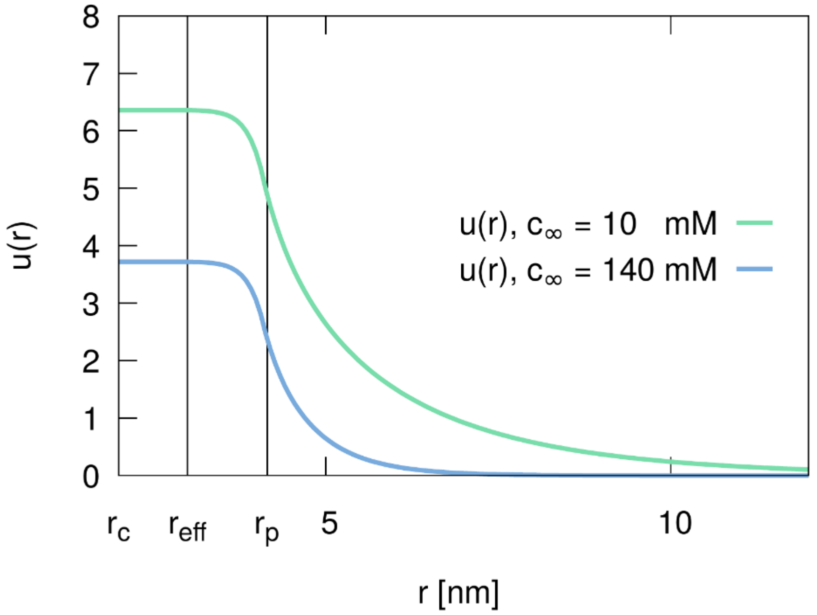

Figure S21: Solutions of the PBE (Equation S5) for different Cl^- bulk concentrations c_∞ (*i.e.* Z=1) for the following parameters: $\tilde{\rho}_{layer}$ = 1.2, r_c = 2 nm, r_{eff} = 3 nm, r_p = 4 nm. The layer boundaries r_c, r_{eff}, and r_p are indicated by vertical lines.

In order to account for different distances r_F - r_p of the Cl- reponsive fluorophore to the NP surface, we used the one-dimensional Edwards equation for calculating the end-to-end distribution G(r_{eff}, r, N) of the PEG polymer spacer with a given number of Kuhn segments N:[19]

$$[\partial_N - \frac{b^2}{2}\partial_r^2]G(r,r',N) = 0 \qquad \text{(Equation S6)}$$

Hereby $b = \langle\vec{b}^2/3\rangle^{1/2}/l_B$ is one component of the root mean squared Kuhn segment size in units of l_B. For sake of simplicity, we assume the NP volume as a planar restriction for the polymer conformations. The polymer is prevented from fully entering into the polymer surface coating by requiring that G(r, r', N) = 0 for $r < r_c$. $r_{eff} - r_c$ is the penetration depth of the PEG into the hydrophobic layer. Note that the thiol-water interface is expected to be subject to fluctuations. Note that layer details are most likely not very realistic, as the PEG should start at the surface of the hydrophilic layer. The end-distribution $P_e(r)$ of the polymer is proportional to G(r_{eff}, r, N). We use a monomer molar weight of 44 g/mol, a number of 4 monomers per Kuhn segment, and a dimensionless Kuhn segment size of b = 0.65 nm $/\sqrt{(3)}$ $/l_B$.

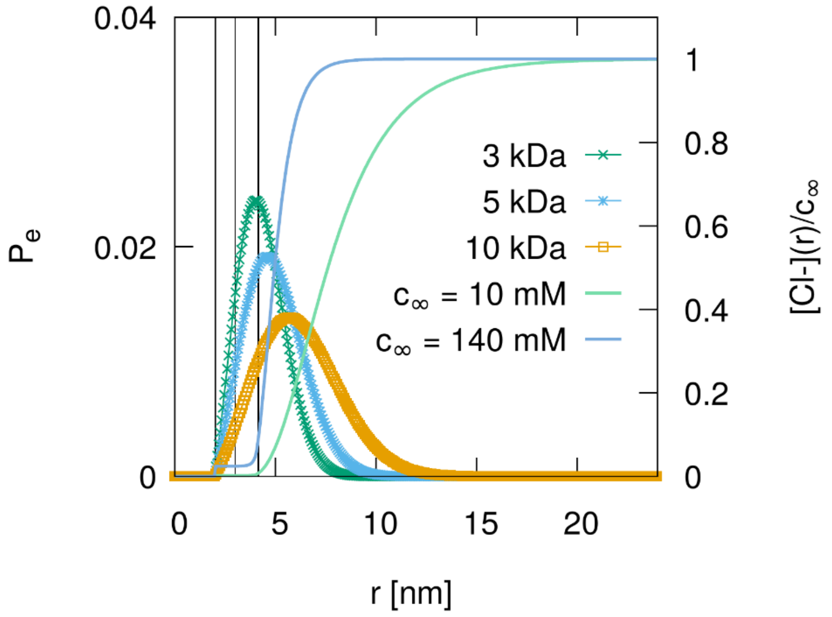

Figure S22: End monomer distributions $P_e(r)$ for PEG chains grafted at $r = r_{eff}$ (center vertical line) of molecular weights 3 kDa, 5 kDa, and 10 kDa. Also, the normalized charge distributions for Z = 1 are also shown for two given molar bulk Cl^- concentrations c_∞. The layer boundaries r_c, r_{eff}, and r_p are indicated by vertical lines from left to right, respectively. The anchor point of the polymer is assumed at ~ r_{eff} in order to obtain a good fit in Figure S23.

Now the results from Figure S21 and Figure S22 can be used to calculate the Cl^- response of the MQAE-modified NPs according to Figure S15. To this end, first, the Cl^--response of free MQAE needs to be taken into account. Using the following calibration curve (c_∞ corresponds to $c(Cl^-)$ in Figure S15)

$$I(c_\infty) = exp(-c_\infty^{0.521586}/0.107639\ mol/L) + 0.347651\ L/\text{mol} \cdot c_\infty \qquad \text{(Equation S7)}$$

a relatively good agreement of the theoretical prediction <I> with the experimental intensities shown in Figure S15 is obtained, when applying Equation S3 to Equation S7, taking into account the solution of Equation S6.[20] With this, the renormalized Cl^--concentrations resulting from the PBE can be calculated in dependence of the used PEG spacers. Results are shown in Figure S23. Both, experimental and theoretical results demonstrate consistently how the increase of Cl^- concentration can be quantitatively mapped to a characteristic increase of Cl^--induced quenching of the end-grafted fluorophore MQAE. The comparison between the experimental and theoretical curves in Figure S23 shows that the theoretical estimates for the intensities systematically overestimate the experimental results for smaller concentrations. Possible origins can be specific and Cl^--dependent interactions of polymer and fluorophore with the thiol-water interface, variations of the dielectric constant near the NP interface, that could be included into a model in the future.

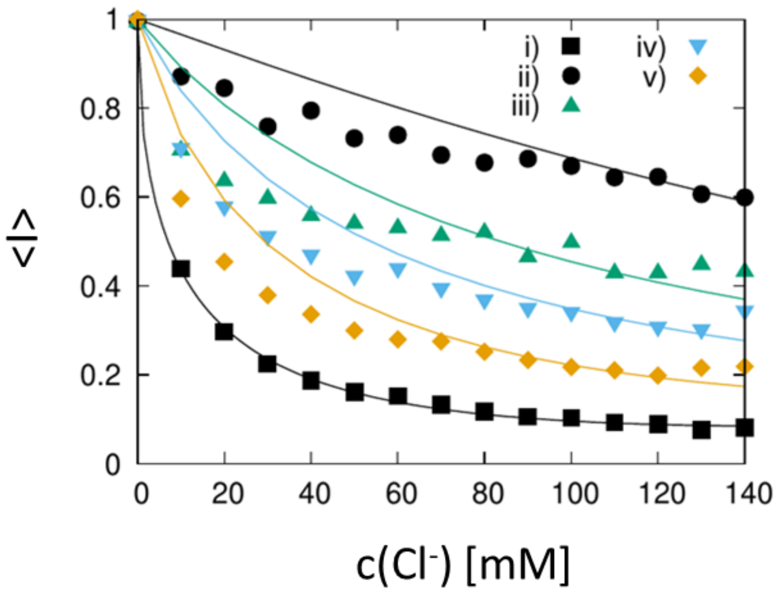

Figure S23: Experimental intensities (closed symbols) for Z=1 and expected intensities (lines, as calculated with Equation S3) for various spacer lengths between 0 and 10 kDa. Symbols and line labels i) … v) are identical to Figure S15, *i.e.* i) denotes the calibration curve (Equation S7) compared to free bulk measurement, ii) corresponds to a fluorescent probe located at r_{eff} + 0.5 nm, and iii) to v) show the estimated response for 3 kDa, 5 kDa, and 10 kDa spacers, respectively.

4 References

(1) Brust, M.; Fink, J.; Bethell, D.; Schiffrin, D. J.; Kienly, C. Synthesis and Reactions of Functionalized Gold Nanoparticles. *J. Chem. Soc., Chem. Commun.* **1995**, 1655-1656.

(2) Geidel, C.; Schmachtel, S.; Riedinger, A.; Pfeiffer, C.; Müllen, K.; Klapper, M.; Parak, W. J. A General Synthetic Approach for Obtaining Cationic and Anionic Inorganic Nanoparticles *via* Encapsulation in Amphiphilic Copolymers. *Small* **2011**, *7*, 2929–2934.

(3) Hühn, J.; Carrillo-Carrion, C.; Soliman, M. G.; Pfeiffer, C.; Valdeperez, D.; Masood, A.; Chakraborty, I.; Zhu, L.; Gallego, M.; Zhao, Y.; Carril, M.; Feliu, N.; Escudero, A.; Alkilany, A. M.; Pelaz, B.; Pino, P. d.; Parak, W. J. Selected Standard Protocols for the Synthesis, Phase Transfer, and Characterization of Inorganic Colloidal Nanoparticles. *Chemistry of Materials* **2017**, *29*, 399−461.

(4) Pellegrino, T.; Manna, L.; Kudera, S.; Liedl, T.; Koktysh, D.; Rogach, A. L.; Keller, S.; Rädler, J.; Natile, G.; Parak, W. J. Hydrophobic Nanocrystals Coated with an Amphiphilic Polymer Shell: A General Route to Water Soluble Nanocrystals. *Nano Letters* **2004**, *4*, 703-707.

(5) Lin, C.-A. J.; Sperling, R. A.; Li, J. K.; Yang, T.-Y.; Li, P.-Y.; Zanella, M.; Chang, W. H.; Parak, W. J. Design of an Amphiphilic Polymer for Nanoparticle Coating and Functionalization. *Small* **2008**, *4*, 334-341.

(6) Sperling, R. A.; Liedl, T.; Duhr, S.; Kudera, S.; Zanella, M.; Lin, C.-A. J.; Chang, W. H.; Braun, D.; Parak, W. J. Size Determination of (Bio-) Conjugated Water-Soluble Colloidal Nanoparticles: A Comparison of Different Techniques. *J. Phys. Chem. C* **2007**, *111*, 11552-11559.

(7) Liu, X. O.; Atwater, M.; Wang, J. H.; Huo, Q. Extinction Coefficient of Gold Nanoparticles with Different Sizes and Different Capping Ligands. *Colloids Surf., B* **2007**, *58*, 3-7.

(8) Riedinger, A.; Zhang, F.; Dommershausen, F.; Röcker, C.; Brandholt, S.; Nienhaus, G. U.; Koert, U.; Parak, W. J. Ratiometric Optical Sensing of Chloride Ions with Organic Fluorophore - Gold Nanoparticle Hybrids: A Systematic Study of Distance Dependency and the Influence of Surface Charge. *Small* **2010**, *6*, 2590-2597.

(9) Teolato, P.; Rampazzo, E.; Arduini, M.; Mancin, F.; Tecilla, P.; Tonellato, U. Silica Nanoparticles for Fluorescence Sensing of Zn-II: Exploring the Covalent Strategy. *Chemistry--A European Journal* **2007**, *13*, 2238-2245.

(10) Zhang, F.; Ali, Z.; Amin, F.; Feltz, A.; Oheim, M.; Parak, W. J. Ion and pH sensing with colloidal nanoparticles: influence of surface charge on sensing and colloidal properties. *ChemPhysChem* **2010**, *11*, 730-735.

(11) Zhang, F.; Lees, E.; Amin, F.; Rivera_Gil, P.; Yang, F.; Mulvaney, P.; Parak, W. J. Polymer-Coated Nanoparticles: A Universal Tool for Biolabelling Experiments. *Small* **2011**, *7*, 3113-3127.

(12) Pellegrino, T.; Kudera, S.; Liedl, T.; Javier, A. M.; Manna, L.; Parak, W. J. On the Development of Colloidal Nanoparticles towards Multifunctional Structures and their Possible Use for Biological Applications. *Small* **2005**, *1*, 48-63.

(13) Rivera Gil, P.; Jimenez de Aberasturi, D.; Wulf, V.; Pelaz, B.; del Pino, P.; Zhao, Y.; de la Fuente, J.; Ruiz de Larramendi, I.; Rojo, T.; Liang, X.-J.; Parak, W. J. The Challenge to Relate the Physicochemical Properties of Colloidal Nanoparticles to Their Cytotoxicity. *Accounts of Chemical Research* **2013**, *46*, 743-749.

(14) Hühn, D.; Kantner, K.; Geidel, C.; Brandholt, S.; De Cock, I.; Soenen, S. J. H.; Rivera Gil, P.; Montenegro, J.-M.; Braeckmans, K.; Müllen, K.; Nienhaus , G. U.; Klapper, M.; Parak, W. J. Polymer-Coated Nanoparticles Interacting with Proteins and Cells: Focusing on the Sign of the Net Charge. *ACS Nano* **2013**, *7*, 3253-3263.

(15) Fernández-Argüelles, M. T.; Yakovlev, A.; Sperling, R. A.; Luccardini, C.; Gaillard, S.; Medel, A. S.; Mallet, J.-M.; Brochon, J.-C.; Feltz, A.; Oheim, M.; Parak, W. J. Synthesis and Characterization of Polymer-Coated Quantum Dots with Integrated Acceptor Dyes as FRET-based Nanoprobes. *Nano Letters* **2007**, *7*, 2613-2617.

(16) Pellegrino, T.; Sperling, R. A.; Alivisatos, A. P.; Parak, W. J. Gelelectrophoresis of Gold-DNA Nanoconjugates. *Journal of Biomedicine and Biotechnology* **2007**, *2007*, 26796.

(17) Sperling, R. A.; Pellegrino, T.; Li, J. K.; Chang, W. H.; Parak, W. J. Electrophoretic Separation of Nanoparticles with a Discrete Number of Functional Groups. *Advanced Functional Materials* **2006**, *16*, 943-948.

(18) Fixman, M. The Poisson–Boltzmann Equation and Its Application to Polyelectrolytes. *J. Chem. Phys.* **1979**, *70*, 4995–5005.

(19) Doi, M.; Edwards, S. F.: The Theory of Polymer Dynamics. Oxford University Press: Oxford, 1994.

(20) Huang, B.; Baulin, V.A. IPEC Solver: Numerical simulation tool to study inter-polyelectrolyte complexation. Comput. Phys. Commun. **2013**, *184*, 2221–2229.
